# P-Type Metal Oxide Semiconductor Thin Films: Synthesis and Chemical Sensor Applications

**DOI:** 10.3390/s22041359

**Published:** 2022-02-10

**Authors:** Abderrahim Moumen, Gayan C. W. Kumarage, Elisabetta Comini

**Affiliations:** Sensor Laboratory, Department of Information Engineering, University of Brescia, Valotti 9, 25123 Brescia, Italy; a.moumen@unibs.it (A.M.); g.wadumasthree@unibs.it (G.C.W.K.)

**Keywords:** p-type metal-oxide semiconductors, thin films, synthesis techniques, PVD, CVD, liquid-phase route, chemical sensors

## Abstract

This review focuses on the synthesis of p-type metal-oxide (p-type MOX) semiconductor thin films, such as CuO, NiO, Co_3_O_4_, and Cr_2_O_3_, used for chemical-sensing applications. P-type MOX thin films exhibit several advantages over n-type MOX, including a higher catalytic effect, low humidity dependence, and improved recovery speed. However, the sensing performance of CuO, NiO, Co_3_O_4_, and Cr_2_O_3_ thin films is strongly related to the intrinsic physicochemical properties of the material and the thickness of these MOX thin films. The latter is heavily dependent on synthesis techniques. Many techniques used for growing p-MOX thin films are reviewed herein. Physical vapor-deposition techniques (PVD), such as magnetron sputtering, thermal evaporation, thermal oxidation, and molecular-beam epitaxial (MBE) growth were investigated, along with chemical vapor deposition (CVD). Liquid-phase routes, including sol–gel-assisted dip-and-spin coating, spray pyrolysis, and electrodeposition, are also discussed. A review of each technique, as well as factors that affect the physicochemical properties of p-type MOX thin films, such as morphology, crystallinity, defects, and grain size, is presented. The sensing mechanism describing the surface reaction of gases with MOX is also discussed. The sensing characteristics of CuO, NiO, Co_3_O_4_, and Cr_2_O_3_ thin films, including their response, sensor kinetics, stability, selectivity, and repeatability are reviewed. Different chemical compounds, including reducing gases (such as volatile organic compounds (VOCs), H_2_, and NH_3_) and oxidizing gases, such as CO_2_, NO_2_, and O_3_, were analyzed. Bulk doping, surface decoration, and heterostructures are some of the strategies for improving the sensing capabilities of the suggested pristine p-type MOX thin films. Future trends to overcome the challenges of p-type MOX thin-film chemical sensors are also presented.

## 1. Introduction

In recent years, emergency scenarios have led to increased demand for high-performance chemical gas sensors due to industrialization and population growth [1]. Currently, chemical gas sensors are primarily used to improve and save human life, and this is expected to remain the case for the foreseeable future. Chemical gas sensors are used in a variety of applications, ranging from environmental protection, food and water safety, and security to medical diagnosis [1,2,3]. Chemical sensors, and gas sensors in particular, are devices that convert chemical information, such as chemical or gas presence (input), into an exploitable signal (output). Among the various chemical gas sensors that have been investigated in recent years, semiconducting metal-oxide conductometric sensors have shown impressive sensing performances. Low production costs, miniaturization capabilities, fast response and recovery time, and high response to low concentrations of chemicals are just a few of the advantages, whereas selectivity is one of its limitations [4]. The principle behind the operation of such devices is based on the surface interaction that occurs between the sensing material (MOX semiconductor) and the gas [5]. In particular, the measurable interaction is a surface reaction caused by adsorption–desorption, which leads to a change in the electrical conductance (or resistance) of MOX as a result of the charge-carrier transfer [6]. The surface reaction of MOX sensors is driven by many factors, such as semiconducting nature (n-type or p-type), target gas (reducing or oxidizing gas), size and morphology, point defects, and MOX compositions. MOX can be categorized into two types: n-type MOX and p-type MOX. N-type MOX have oxygen-vacancy (V_O_) donors responsible for n-type conductivity, and the majority of charge carriers are electrons ($e^{-}$) On the other hand, p-type semiconductors are metal-ion-deficient, and the majority of charge carriers are holes (h+) [1,7].

In contrast to p-type MOX, which account for only 10% of MOX [8], n-type MOX are the most investigated semiconductors. SnO_2_, ZnO, TiO_2_, and WO_3_ are the most common MOX for fabricating conductometric gas sensors due to their high chemical and thermal stability relative to other MOX [9]. Among them, SnO_2_ and ZnO thin films are the most investigated. Indeed, p-type MOX, such as CuO, NiO, Cr_3_O_4_, and Co_3_O_4_ exhibit important advantages, such as low humidity dependency, high chemical stability, and excellent catalytic properties, which make them ideal catalysts for improving the performance of conductometric gas sensors. Thus, researchers have concentrated on developing p-type MOX thin films and have been working on building up highly efficient gas sensors [3,9]. This review will provide insight into p-type MOX semiconductor conductometric chemical sensors. Reports published on p-type MOX thin films have covered many applications, considering the different gases detected, low and high concentrations, as well as humid and dry environments. Furthermore, the sensors have demonstrated detection capability at room temperature (RT), opening up the possibility of portable applications for p-type MOX gas sensors [8,10]. Due to their advantages, p-type MOX sensors using thick film materials are available on the market [11].

MOX thin films have several advantages over thick films, including enhanced surface effects, high specific areas, and high porosity. As a result of these properties, thin films are ideal for a variety of applications, including optoelectronic devices, catalyst nanomedicine, and, in particular, chemical sensors. The choice of an appropriate thin-film synthesis method has a significant impact on the efficiency of MOX materials. In general, top-down and bottom-up approaches are the global approaches used to describe the synthesis route for thin-film materials. These approaches involve several sub-routes, including liquid, vapor, and solid phases [12]. However, the composition, defects, morphology, sizes, and thickness of MOX thin films influence the performance of chemical gas sensors. The synthesis technique, however, is capable of controlling the overall properties of p-type MOX [12].

This review investigates several methods for growing p-type MOX, including magnetron sputtering, thermal oxidation, thermal evaporation, molecular-beam-epitaxy (MBE), chemical vapor deposition (CVD), sol–gel-assisted by dip-and-spin coating, spray pyrolysis, and electrodeposition. This study examines the growth mechanisms of each technique, taking into account a number of experimental parameters and thermodynamic factors that impact the growth of p-type MOX. Furthermore, the sensing mechanism of p-type MOX is explored. In the final section, we will examine the performance of different p-type MOX, mainly CuO, NiO, Co_3_O_4_, and Cr_2_O_3_, as well as functionalized, doped, and composite structures. In this context, many reducing gases are considered, such as volatile organic compounds (VOCs) (such as acetone and ethanol), hydrogen, and NH_3_, as well as oxidizing gases, such as NO_2_, CO_2_, and O_3_.

## 2. Mechanism of P-Type MOX Thin Films

**Figure 1:**
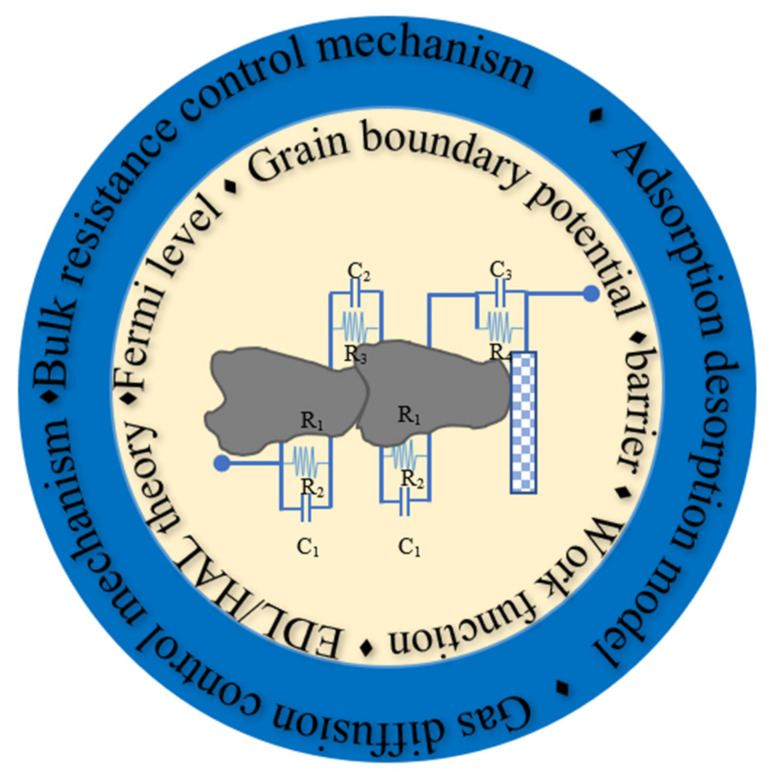
Schematics of the sensing-mechanism categories.

The chemical-sensing mechanism can be divided into chemical interaction and electrical transduction, as illustrated in Figure 1 [13]. The first deals with the interaction between gases and materials (adsorption/desorption, gas-diffusion control mechanisms, and bulk-resistance control mechanism) [13], whereas the second concerns changes in electrical properties from a microscopic perspective (grain-boundary barrier-control theory, Fermi-level control theory, and electric-double-layer (EDL) theory). Changes in electrical conductance upon chemical/gas interaction on the semiconducting sensing layer will be discussed in the second section.

In conductometric MOX gas sensors, gas detection is based on the change in electrical conductance of the sensing element in response to exposure to gas. Adsorption of gases on the surface of MOX results in the transfer of carrier charges between the gas and the MOX, which permits tuning of the electrical conductance, depending on the type of majority carriers on the MOX and the nature of the interacting gas (oxidizing or reducing). For instance, when interacting with an oxidizing gas, the conductivity of p-type MOX increases; conversely, reducing gases decrease the conductivity of p-type MOX. Accordingly, ionosorbed species play an important role in electrical conduction. As MOX are exposed to air, oxygen is ionosorbed, which dramatically alters the sensor conductance. Usually, oxygen can adsorb in three forms: molecular $O_{2}^{-}$, atomic $O^{-}$ and $O^{2-}$ species at temperatures of <150 ℃, 150–400 ℃, and >400 ℃, respectively, as shown in Equations (1)–(3) [8]. Once oxygen ions are chemisorbed, the concentration of free charge carriers (holes) increases in p-type materials. Chemisorbed oxygen species cause electron trapping from the MOX valence band, which leads to a hole-accumulating layer (HAL) near the semiconductor surface [14]. This phenomenon can be explained in detail with the support of a band diagram of semiconductors [14].

**Figure 2:**
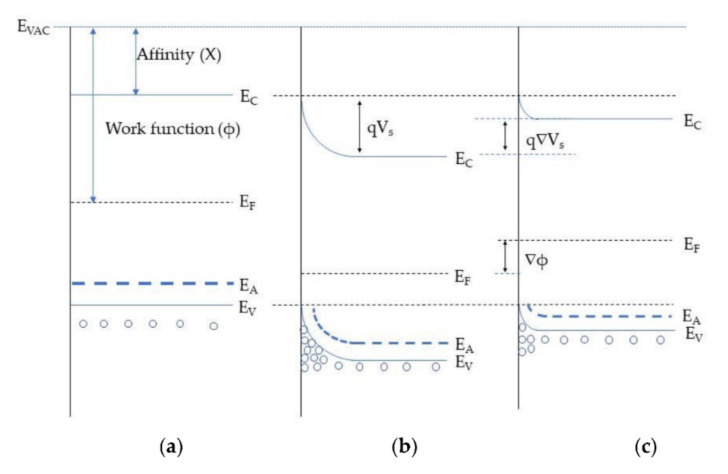
Energy-band structure at the near surface when interacting with oxygen and reducing gases: (**a**) Prior to any surface interaction. (**b**) Electron trapping and formation of the hole-accumulating layer due to oxygen adsorption. (**c**) The decrease in surface charge due to the interaction with the reducing gas. E_C_ is the conduction-band position; E_F_ is the Fermi-level position; Ev is the valance-band position; q is electron charge; qV_S_ is the potential barrier. Reprinted with permission from [15].

Usually, in a p-type semiconductor, such as CuO, NiO, Co_3_O_4_, and Cr_2_O_3_, the acceptor level is near the valance band. Typically, the acceptor level is fully ionized (filled), leaving holes in the valance band at room temperature (Figure 2a) [15]. Once the metal-oxide surface is exposed to air, oxygen from the air gets adsorbed on the metal-oxide surface by trapping electrons from surface states. The result is band bending and an increase in hole concentration near the interface, forming a hole-accumulating layer at the MOX surface (Figure 2b) [15]. This causes a decrease in the MOX resistance. Consequently, when a reducing gas is introduced, it reacts with adsorbed oxygen and may chemisorb by transferring electrons to the MOX. Accordingly, the recombination of electron holes increases, narrowing the accumulation layer. Hence, its resistance increases, accompanied by a decrease in band bending (qΔV), as shown in Figure 2c. On the other hand, oxidizing gases are considered electron acceptors due to their high electron affinity. The molecules react with the surface of p-type MOX by direct adsorption on the surface or by reaction with pre-adsorbed oxygen. In both cases, as a result of the adsorption of oxidizing gases, electrons are released from the surface, leading to an increase in the width of the HAL, as well as increased conductivity.

However, the charge carriers (holes) in the accumulation layer are restored and cause a decrease (or increase) in sensor conductance based on desorption of this reducing (or oxidizing) molecule from the metal-oxide surface.

On the other hand, humidity plays a significant role in the sensing mechanism. Water $\left(H_{2}O\right)$ molecules dissociate on the metal-oxide surface, resulting in hydroxyl ions $\left({\mathrm{OH}}^{-}\right)$ and $protons\;\left(H^{+}\right)$. Subsequently, the ratio of adsorbed $H_{2}O$ to active sites (in MOX) governs the absorption mechanism, where chemisorption dominates in a low-humidity environment and physisorption dominates in a high-humidity environment. At low humidity levels, $H_{2}O$ molecules chemisorb at the active sites of semiconductor metal-oxide surfaces, resulting in hydroxyl groups and mobile protons. The hydroxyl ions generated by dissociation of $H_{2}O\;$ bind to the metal cations and form rooted hydroxyl groups [16], whereas protons react with oxygen to form secondary hydroxyl ions (${\mathrm{OH}}^{+}$). When the humidity level is high, all active sites are occupied by the first chemisorbed water molecules. In this case, the water molecules physically adsorb and form physiosorbed layers on top of the chemisorbed layer. Additionally, the water molecules in these layers bind with $H^{+}$ to form a large amount of hydronium ($H_{3}O^{+}$) [3]. Accordingly, electrical conductance is caused by proton $H^{+}$ hopping between neighboring water molecules. Such proton conduction affects the baseline resistance. Additionally, at higher humidity levels, water molecules compete with gas molecules, resulting in a reduction in the amount of chemisorbed oxygen species and a reduction in gas-molecule adsorption.

(1) $$O_{2\left(\mathrm{ads}\right)}+e^{-}\to O_{2}^{-}$$

(2) $$O_{2}^{-}+e^{-}\to \;2O^{-}$$

(3) $$2O^{-}\to O^{2-}$$

## 3. Synthesis of P-Type MOX Thin Films

### 3.1. Vapor-Phase Growth Methods

The vapor-phase route includes numerous techniques that enable the nucleation and growth of p-type MOX films, with excellent control of the crystal structure and thickness of the films. A material source (solid, powder, or gaseous) can be evaporated and deposited on a substrate, where nucleation and growth phenomena may occur, depending on the nature of the reaction. Physical vapor deposition (PVD) and chemical vapor deposition (CVD) are part of the vapor-phase growth. PVD involves the condensation of vaporized gaseous molecules of the desired material onto a substrate. Despite high costs, PVD can precisely control the thickness, crystal structure, and microstructure of thin films. It includes several methods, though the most investigated are magnetron sputtering, thermal evaporation, and molecular-beam epitaxy (MBE). Additionally, thermal oxidation is another PVD technique, which uses oxidation rather than condensation to produce MOX thin films. In the case of CVD, a chemical reaction with the gaseous molecules contained in the vapor occurs on the surface of the substrate. CVD includes many methods, such as atmospheric-pressure CVD (APCVD), low-pressure CVD (LPCVD), plasma-assisted CVD (PACVD), and atomic layer deposition (ALD). CVD provides high control over morphology and defects, allows for large-scale production, and promotes the formation of heterojunctions; however, it is adversely affected by the appearance of harmful byproducts.

#### 3.1.1. Magnetron Sputtering

**Figure 3:**
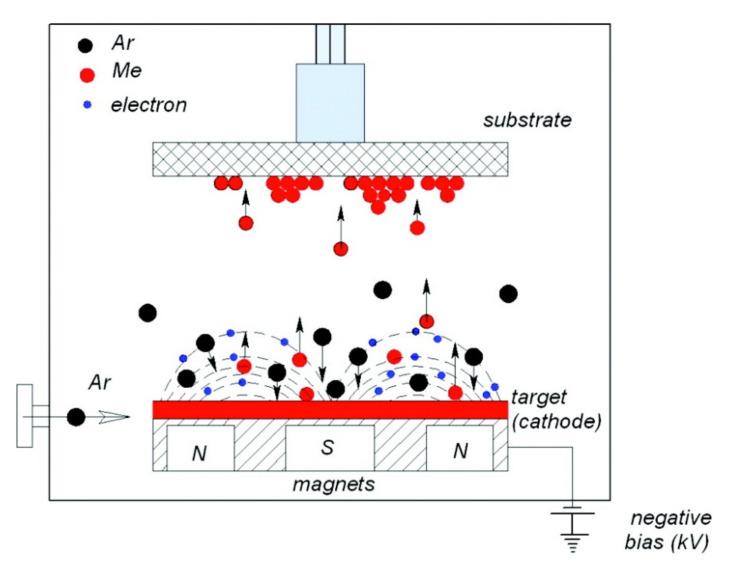
Schematic of magnetron sputtering. Me refers to the metal used as a target, while N and S refer to the poles of the magnetron unit. Reprinted with permission from [17].

Magnetron sputtering is a common PVD technique for synthesis of metal-oxide films, including CuO, NiO, Co_3_O_4_, and Cr_2_O_3_. This technique involves the creation of a plasma by applying a discharge between two electrodes, resulting in the ionization of a plasma gas (usually argon) in a vacuum (Figure 3) [17]. In sputtering, plasma containing positive argon ions bombards a target, and the atoms or aggregates of atoms that are knocked out are accelerated towards the substrate. Sputtering targets can be metal oxides or metals. It is also possible to exploit reactive magnetron sputtering by injecting a reactive gas, such as O_2_ or N_2_. Ar and O_2_ mixtures are generally used for the synthesis of highly stoichiometric metal oxides, whereas Ar and N_2_ mixtures are used for the deposition of metal-nitride coatings [18,19].

Many p-type MOX thin films have been grown using magnetron sputtering. However, the stoichiometry, microstructure, and particle size of MOX films are influenced by several factors, including substrate temperature, gas environment, pressure, and deposition time.

The temperature of the substrate has been shown to have a significant influence on phase formation in CuO. When magnetron sputtering is used to deposit a CuO thin film, the primary concern should be the phase mixtures due to the formation of Cu_2_O and Cu_3_O_4_ during deposition. This issue can be overcome by controlling the oxygen flow with reactive magnetron sputtering or by controlling the substrate temperature [20]. Upon heating the substrate to a temperature below 250 ℃, Cu_2_O (as a dominant phase) and traces of CuO are formed. Deposition at temperatures above 300 ℃ ensures the formation of stable and pure CuO with a monoclinic structure, whereas heating the samples above 450 ℃ leads to a mixture of CuO, Cu_2_O, and Cu_3_O_4_.

Magnetron sputtering has also been used to produce Co_3_O_4_ thin films [21]. Like CuO thin films, the substrate temperature is responsible for tuning the cobalt-oxide phase. It was demonstrated that Co_3_O_4_ with a spinel structure can be formed at temperatures ranging from 300 ℃ to 600 ℃ [22]. The growth of Co_3_O_4_ at high temperatures may be attributed to the fast migration of the atoms in a hot substrate due to the high kinetic energy, which results in high grain-growth speed. However, discharge power was also found to induce the transition from CoO at high power (180 W to 240 W) to Co_3_O_4_ at low power (80 W to 160 W), whereas a mixture of phases was achieved at 170 W [21].

Phase-mixture problems can be overcome by controlling oxygen flow. In situ plasma diagnosis was performed to study the sputtering mechanism, and it was found that the phase transition from Cu_2_O to CuO is governed by oxygen density in reactive magnetron sputtering [23]. The oxygen density tunes the oxidation state of the final metal-oxide thin film.

In the case of a metal target, reactive oxygen species form covalent bonds with metal bonds, resulting in MxOy thin films. Nayan et al. demonstrated that 17 sccm (standard cubic centimeters per minute) is the critical oxygen-flow ratio for the transition to occur, leading to the formation of pure CuO.

Pure Cr_2_O_3_ was grown by sputtering metallic chromium at temperatures above 230 ℃  in a mixture of Ar and O_2_ (varying between 15 and 25%). In this study, high stoichiometry of the films was obtained at high temperatures with a 20% oxygen content [24]. O_2_ pressure also affects surface morphology and roughness. A higher oxygen concentration leads to a more porous structure of thin films. Erdal et al. grew NiO thin films using a magnetron by adjusting the oxygen pressure. According to the authors, as the oxygen pressure increased, a granular surface with higher porosity was achieved [25]. Accordingly, the authors reported high gas-sensing capabilities of NiO towards hydrogen due to the porous structure, providing a high adsorption rate [23]. One of the benefits of reactive magnetron sputtering is that it permits growth at room temperature without the need for additional annealing. Growth at room temperature offers the possibility of using flexible substrates, such as plastic and paper. Gas sensors can be manufactured using these substrates for portable, flexible gas sensors.

#### 3.1.2. Thermal Evaporation

**Figure 4:**
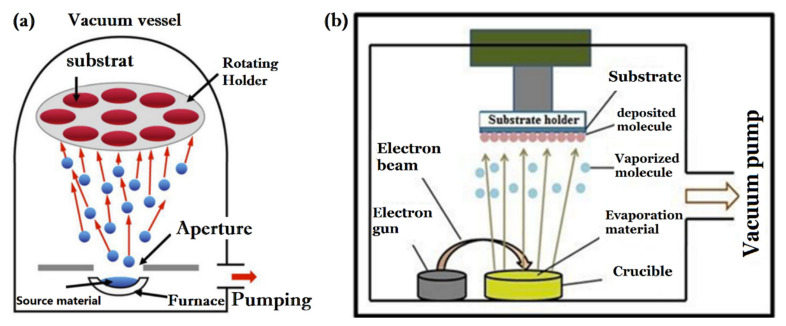
Schematic of thermal evaporation. (**a**) Resistive thermal evaporation. (**b**) Electron-beam evaporation. Reprinted with permission from [27,28].

In the process of deposition by thermal evaporation, atoms of vapor from the source material are condensed on a substrate situated opposite the source material in a vacuum. Deposition of metal-oxide thin films is carried out in three steps: evaporation of the MOX source, transfer of vapor atoms/molecules, and their condensation on the surface of the substrate, forming the thin films. It would be possible to raise the vapor with less contamination using an ultra-high vacuum. The evaporation of source material can, however, be achieved, even at low vapor pressures. Based on the heat source used, two types of thermal evaporation may be distinguished; (1) resistive thermal evaporation, which involves heating the source material by heating the crucible with the Joule effect. The heat can be induced by using a tungsten filament in this case. (2) Electron-beam evaporation, in which a “gun” of the electron is generated by thermionic emission. Through the application of high voltage, the electron beam is then accelerated towards the source of the material, resulting in its evaporation. The electron-beam evaporator has the advantage of being able to reach the higher temperatures required for the evaporation of some materials, as well as a high deposition rate; however, it has safety issues due to high applied voltages. A schematic of thermal evaporation is shown in Figure 4 [26,27].

Thermal evaporation has been used to deposit a variety of p-type thin films, including CuO, NiO, Co_3_O_4_, and Cr_2_O_3_. A traditional method of producing metal-oxide thin films involves evaporating metal targets in a vacuum, followed by annealing to achieve oxidation. By evaporating Cr powder in a high-vacuum chamber, followed by annealing at 300 °C for 3 h in air, a smooth and uniform surface of Cr_2_O_3_ film is achieved [29] CuO thin films have been grown by evaporation of Cu powder, followed by heat treatment at different temperatures. In such cases, the annealing temperature controls the size of particles in the films [30]. The same procedure has been applied to grow NiO [31]. The synthesis of thin films using a single step, without annealing process, has been optimized. NiO has been produced through e-beam evaporation at room temperature [32]. According to Al-Kuhaili et al., temperature has been considered as an important factor in avoiding phase mixtures [33]. In their experiment, Cu_2_O powder was evaporated, deposited on a heated substrate, and then annealed at 500 °C for 3 h to form tenorite CuO. Alternatively, as-deposited samples (unheated substrates) without annealing are characterized by the formation of CuO and Cu_2_O phase mixtures.

#### 3.1.3. Thermal Oxidation

Thermal oxidation is a well-known technique for growing SiO_2_ from a silicon wafer. This technique has been widely used to prepare p-type thin-film semiconductors, especially NiO and CuO thin films. In contrast, very few studies have been conducted for the preparation of Co_3_O_4_ and Cr_2_O_4_ thin films due to the different oxidation states that can occur during the oxidation of Co and Cr metals. Thermal oxidation has many advantages, including low production costs, large-scale production, and the absence of hazardous chemicals. However, there are drawbacks from the industrial perspective due to the high energy consumption associated with the elevated oxidation temperature.

Metals can be oxidized by thermal oxidation to form MOX thin films. It is important to note that magnetron sputtering and thermal evaporation are generally used for preparing the metal layer necessary for thermal oxidation. Several substrates have been used for the growth of MOX thin films by thermal oxidation, including glass and Silicon. Cu and Ni metals have been deposited on SiO2/Si substrate at room temperature by evaporating Cu and Ni, using a vacuum evaporator. Oxidation is accomplished by annealing in an air atmosphere inside a tubular furnace [34,35]. CuO was grown on glass substrates using the above procedure [36]. NiO thin films were grown using thermal oxidation, and Ni metal was deposited using magnetron sputtering on quartz substrates at ambient conditions [37].

Briefly, thermal oxidation can be achieved in a furnace operating at high temperatures, in a controlled oxygen atmosphere or even in the air (dry and wet oxidation). The metal films are oxidized in air using an alumina or quartz tube that is heated up at high temperatures (up to 1100 °C). The annealing treatment, including annealing temperature and annealing time, is a crucial factor that can control stoichiometry, oxidation state, surface morphology, electrical and optical properties, as well as defects in p-type MOX thin films [38].

**Figure 5:**
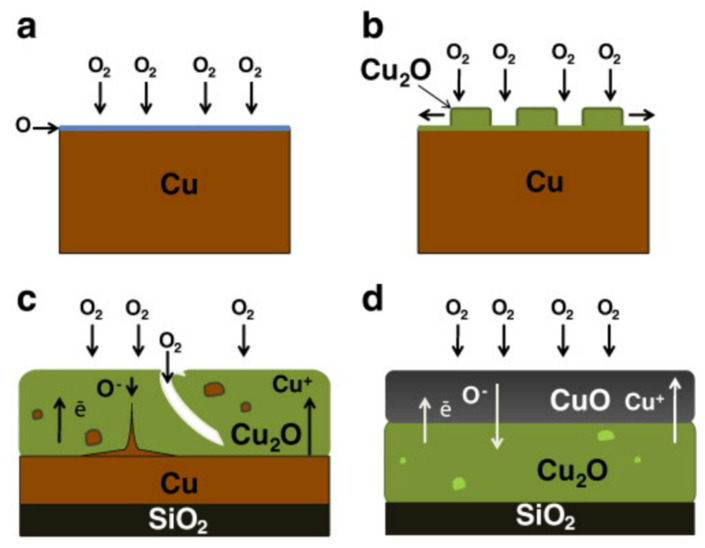
The growth mechanism of CuO thin films using thermal oxidation. (**a**) Chemical adsorption of oxygen on the cooper surface and formation of the oxide layer. (**b**) Nucleation and formation of Cu_2_O on the top of the Cu surface. (**c**) Cu ions diffuse and ionize oxygen atoms, which are then incorporated into the oxide network. Consequently, new oxide layers are formed at the interface oxide/oxygen, and the thickness of Cu_2_O is increased. (**d**) Transformation of Cu_2_O into CuO at a high annealing temperature (complete oxidation). Reprinted with permission from [34].

To better understand the annealing effect, CuO could be a good example. The oxidation mechanism of CuO thin films is shown in Figure 5. The oxidation of CuO thin films from Cu metal is driven by the annealing treatment. In [34], Cu was deposited on SiO_2_/Si substrate at room temperature using an evaporator, and oxidation was achieved in an air atmosphere using a tubular furnace. The effect of annealing treatment at different temperatures from 150 °C to 1000 °C was investigated. At low annealing temperature (250 °C), a Cu_2_O phase was detected, and when the annealing temperature was increased (300 °C to 1000 °C), CuO formed. Essentially, the mechanism involves the chemical adsorption of oxygen atoms from air to form a Cu_2_O layer that covers the Cu metal layer. Afterward, the nucleation of copper oxide can be extended laterally by increasing the annealing temperature, which will consume the copper metal layer totally (by accelerating the adsorption) and lead to the formation of pure Cu_2_O. However, due to the absence of Cu and a further increase in temperature, pure CuO formed at 1000 °C as a result of the reaction (Cu_2_O + 1/2O_2_ → 2CuO) [34].

Concerning NiO, its thermal oxidation was investigated by varying the annealing temperature [37], and a full oxidation of NiO was achieved at temperatures above 500 °C, with improved NiO crystallinity by further increasing the oxidation temperature. Full oxidation can be reached at 700 °C [37]. Similar to CuO, increasing the annealing accelerates the diffusion of Ni ions from the buffer layer into the NiO layer. In this way, an additional oxide layer is formed until complete oxidation is achieved. The thermal-oxidation mechanism has been explained in many reports [34,35,39]. Another often overlooked factor is the oxidation time. Indeed, this factor could affect the crystallinity of thin films [36].

On the other hand, the durability of thin films is determined by microstructure effects, such as stress, strain, and dislocation. An advanced microstructure study was conducted on Cr_2_O_3_ thin films prepared through thermal oxidation, such as strain relaxation. In this context, an in situ synchrotron radiation measurement was applied in order to determine the strain/stress evolution when low-temperature jumps (of 100 °C) occurred during thermal oxidation from 1000 °C to 700 °C [39]. The objective of this study was to examine the creep rate of thin films, which is strongly related to material stress and microstructure in order to achieve a better quality and durability of MOX thin films.

#### 3.1.4. Molecular-Beam Epitaxy (MEB)

**Figure 6:**
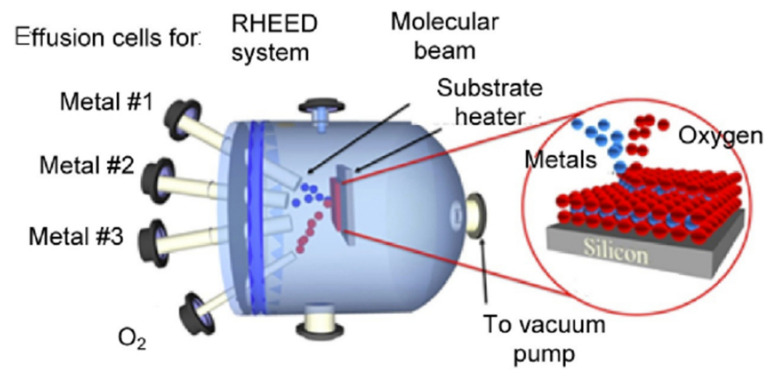
Schematic of MBE technique. Reprinted with permission from [40].

MBE is another method for depositing metal-oxide thin films, especially in the industrial sector. Despite its high cost, slow growth rate, and technical challenges, MBE has been described as an advanced, clean deposition technique that provides layer-by-layer deposition and precise control of the thickness of thin films. The ultra-high vacuum ensures extremely pure growth of thin films. However, MBE is well-known for growing single crystals. MEB is made up of effusion cells, where molecular beams of the elements are generated by evaporation or sublimation (Figure 6) [40]. The process involves the direct impingement of the physical vapor generated from each cell onto the heated substrate, which then leads to condensation, nucleation, and the formation of a single crystal layer.

The growth depends mainly on the nature of the substrate and its temperature. The growth of crystals is usually assisted by in situ analysis using reflection high-energy electron diffraction (RHEED), which is used to find and examine the orientation relationship between the film and its substrate. Importantly, there is no vapor-phase diffusion in UHV MBE, meaning that there is no interaction between the vapor beams due to their long mean free paths. Thus, simple surface interactions occur on the heated substrate, in contrast to some CVD techniques, such as MOCVD.

Many p-type MOX thin films have been grown using MBE, including CuO, NiO, and Co_3_O_4_ thin films. Co_3_O_4_ has been grown on a variety of substrates. Among these substrates, MgAl_2_O_4_ (110) is suitable for epitaxial growth of Co_3_O_4_ in (110) orientation because of the small lattice mismatch [41,42], whereas α-Al_2_O_3_(0001) is also a good choice for (111)-oriented Co_3_O_4_ [43]. The atomic elemental flux and substrate temperature have a significant impact on the final morphology and stoichiometry. In this context, oxygen-assisted MBE has been used to epitaxially grow Co_3_O_4_ (111) thin film on Al_2_O_3_ (0001) single-crystal wafers [43].

Oxygen flux and post-annealing temperature were used to control the oxidation, the purity of cobalt oxide, crystallinity, and, most importantly, the interface between the material and the substrate. Post-annealing has been demonstrated to yield a uniform and smooth surface without changing the stoichiometry of Co_3_O_4_. Post-annealing may also affect the interface structure of Al_2_O_3_ and Co_3_O_4_ due to interdiffusion phenomena. However, these results are not consistent in the case of using MgAl_2_O_4_ (110) substrate, where a stable surface was observed for as- and post-annealed Co_3_O_4_/MgAl_2_O_4_ (110). On the other hand, epitaxial growth of CuO was achieved on (001)MgO, as reported by Kawaguchi et al. [44]. In their research, they found that the elemental flux ratio controls the growth and oxidation of CuO. The ion beam containing O+ was injected into the UHV vacuum chamber. High amounts of oxygen content resulted in the formation of a mixture of CuO and Cu_2_O, while a low amount resulted in a mixture of Cu_2_O and Cu. In this work, controlling the oxygen content in (metal/oxygen ratio) directly influenced the growth of thin films. We can assume that contrary to explanations given previously for Co_3_O4, lattice mismatch is not the predominant factor controlling epitaxial growth. Here, it was the surface energy that determined the orientation of MBE growth. On the other hand, epitaxial growth of NiO(111) on GaN(110) substrate was achieved using radiofrequency (RF)-plasma-assisted MEB with an oxygen-rich growth environment [45]. A detailed explanation can be found in [46].

#### 3.1.5. Chemical Vapor Deposition (CVD)

**Figure 7:**
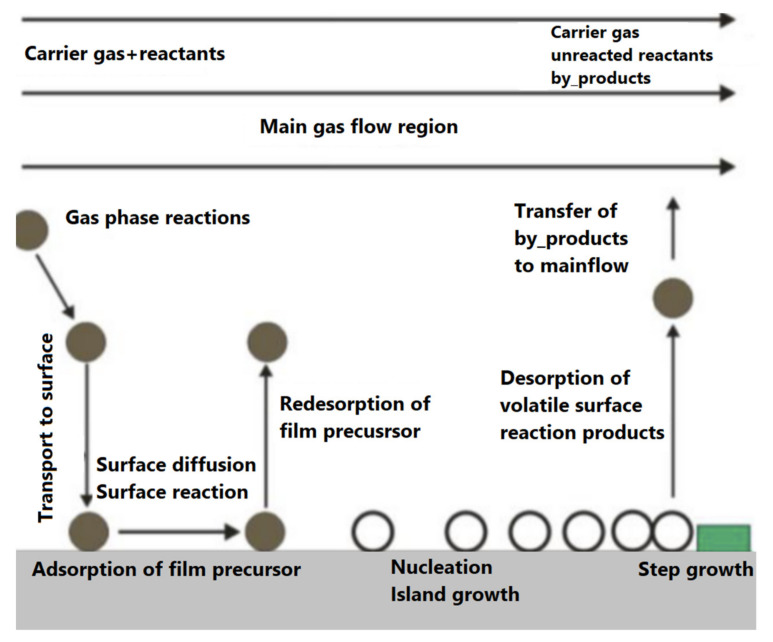
Schematic of the growth mechanism of CVD. Reprinted with permission from [47].

The growth of thin films occurs through a chemical reaction, as opposed to PVD, which uses sputtering or evaporation to condense molecules. CVD relies on chemical reactions occurring within a reaction chamber. The precursors are vaporized inside the reaction chamber at controlled temperatures and pressures, usually under vacuum. Essentially, the elements or reagents (precursors) are volatile molecules in the form of vapor. It is possible for chemical reactions to occur between the precursors themselves or on the surface of the substrate. A carrier gas transports the vapor over the heated surface, inducing nucleation and diffusion and resulting in a high-performance coating. The CVD mechanism is described in Figure 7 [47]. CVD deposition requires a tubular furnace, vapor precursors, and a carrier gas. The growth should be governed by temperature and pressure inside the reaction chamber. A chemical reaction is driven by thermodynamic factors, such as the kinetic energy required for the reaction and Gibbs free energy needed to be reduced to initiate the reaction at the correct temperature and pressure. Many different processes are involved in CVD, such as atmospheric pressure CVD (APCVD), low-pressure CVD (LPCVD) (when the pressure is less than ambient), laser- and photon-assisted CVD, plasma-enhanced CVD (PECVD) (when plasma enhances the decomposition), pulsed CVD, and so-called “atomic-layer deposition” (ALD) [12,48].

Although LPCVD has many advantages over APCVD when it comes to purity and excellent reliability and homogeneity of deposition, it suffers from its slow rate and high-temperature requirement. PECVD may eliminate the need for high temperatures by allowing the reaction to take place at room temperature instead (low-pressure CVD and plasma-enhanced CVD). Lasers or photons are typically used to assist in the decomposition of precursors. As an alternative, ALD allows for layer-by-layer deposition of layers with highly controllable epitaxial thicknesses; however, it suffers from a slow growth rate. Out of several CVD techniques, few of them will be investigated in this review. CVD is widely used to synthesize a variety of nanomaterials. In particular, two-dimensional nanostructures, including graphene and 2D metal dichalcogenides, have been extensively studied. Moreover, besides the advantages of CVD techniques, such as large-scale production, high productivity, cost control, and perfect surface coverage, this route suffers from the generation of hazardous byproducts during the chemical reaction and low control of thickness. As a result, safety precautions must be taken when venting and releasing by-products [12]. CVD techniques have been used to grow many p-type MOX thin films, including CuO, NiO, Cr_2_O_3_, and Co_3_O_4_ [49].

CVD can deposit metal oxides on a variety of substrates, including SiO_2_, Al_2_O_3_, and ITO [50,51]. However, growth is influenced by factors such as substrate temperature. Substrate temperature greatly affects the diffusion and tunes the final morphology of crystalline thin films. In this context, CVD was utilized to prepare CuO thin films by vaporizing Cu(acac)_2_ precursor on a glass substrate heated to temperatures ranging between 350 °C and 650 °C [49]. Tenorite CuO was formed at all temperatures; however, the highest temperatures produced thin films with smooth surfaces and large grains with high crystallinity. High temperatures increased diffusion rates significantly, since a high kinetic energy was available for the adatoms to diffuse; as a result, crystal growth was enhanced [49].

The effect of substrate temperature on the growth of Co_3_O_4_ has been investigated using LPCVD on ITO [51]. Copper oxide grown by PECVD demonstrates a phase transition when the substrate temperature is adjusted [52]. This shows that regardless of the technique used, growth is highly influenced by substrate temperature. P-type MOX thin films, such as Cr_2_O_3_, can be grown at RT using the CVD method [53]. They may also be capable of growing films on flexible substrates, such as plastic and carton, allowing for fabrication of flexible chemical sensors, as reported in the PVD section. Using laser-assisted CVD, Cr_2_O_3_ thin films were grown at RT by photodissociation of Cr(CO)_6_ precursors in an atmosphere containing oxygen and argon [54]. Here, the laser was used to drive the chemical reaction. This research examined the effects of both the laser and the oxygen content. It is necessary to combine both factors in order to grow stoichiometric chrome oxide. The photodissociation of the Cr(CO)_6_ to form Chromium oxide is quite complex and still under discussion [53]. In the presence of selective laser fluence, the Cr(CO)_6_ molecule decomposes into Cr and 6CO, and then to Cr* and 4Co at even higher laser fluences (greater than 50 mJ cm^−2^). Afterward, the photoproducts react with oxygen to form CrO_2_, and then Cr_2_O_3_ can be grown by surface reaction on the surface substrate (2CrO_2_ → Cr_2_O_3_ + 1/2O_2_).

Recent trends in CVD are focused on using metal-organic precursors, which are referred to as MOCVD. For example, epitaxial Co_3_O_4_ (1 1 1) thin films have been produced using MOCVD. Cr_2_O_3_ thin films were prepared using (MO)CVD for anti-wear protection [55]. In fact, even with the issue of precursor stability, MOCVD has a fast growth rate and provides a high level of control over film stoichiometry [56]. As a way to reduce the temperature, plasma-assisted MOCVD (PA-MOCVD) has been employed to produce a stoichiometric NiO phase [57]. Metal-oxide growth through MOCVD is still a challenge from a chemistry perspective, as the precursors may be volatile and even carbon-rich [57,58].

### 3.2. Liquid-Phase Route

As explained before, p-MOX thin films can be fabricated using different routes. However, the liquid-phase route has emerged as the most competitive candidate when it comes to cost effectiveness, large-area electronics, and mass production [59]. In their simplest form, the solutions are prepared by mixing the metal-oxide precursors with a suitable solvent to a known viscosity and composition. Various types of catalysts or inclusions may be added to the solution to give better control over the dissolution of the precursor with the solvent. Precursors can range from simple metal-oxide powders to acetate, chloride, sulphate, nitrate, and their mixtures. They are generally used for preparing colloidal-like sol–gels or liquid precursors containing p-MOX complexes encapsulated by organics, such as those in alkoxide and carboxylate materials. This facilitates the formation of the deposit through formation reactions, such as hydrolysis or condensation processes [59]. This review will report a few procedures widely used to prepare p-type MOX thin films by liquid-phase methods, including sol–gel-assisted dip coating and spin coating, spray pyrolysis, and electrodeposition techniques.

#### 3.2.1. Sol–Gel

**Figure 8:**
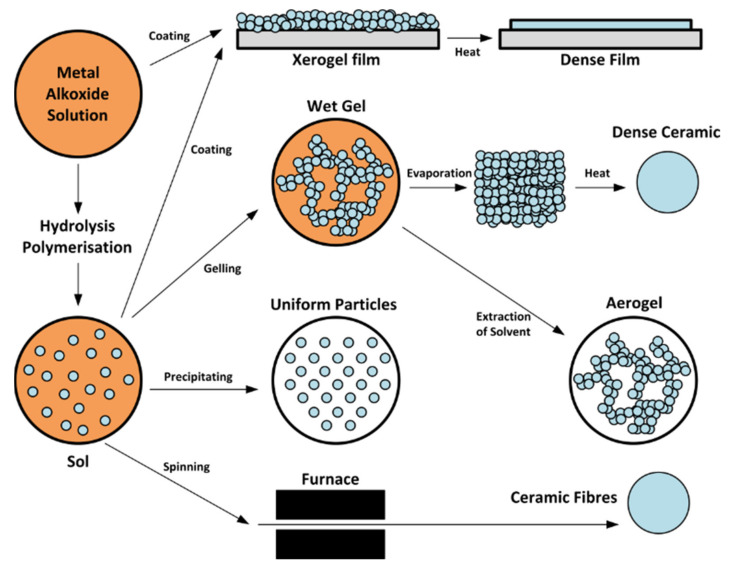
Schematic representation of different stages of the sol–gel process. Taken from [61].

In the “sol–gel” process, the “sol” (or solution) progresses gradually towards the formation of a gel-like “gel” network containing both a liquid phase and a solid phase. Usually, the formation of a sol occurs through hydrolysis and condensation of metal alkoxide precursors. However, a sol can be more generally defined as a colloidal suspension that covers a wider range of systems. According to the International Union of Pure and Applied Chemistry (IUPAC), a colloidal system is a dispersion of one phase in another where, “the molecules or poly-molecular particles dispersed in a medium have at least in one direction a dimension roughly between 1 nm and 1 μm” [60]. In a general sense, the precursor can be either deposited by casting into a suitable container with the desired shape or on a substrate to form a film. This typical sol–gel chemistry is still one of the most extensively employed and studied because of low-cost processing, uniform and homogeneous film formation over large areas, the possibility of a precise stoichiometry, and thickness control. Figure 8 shows a schematic of the different stages of the sol–gel processing method [61].Sol–gel is a simple, low-cost method to produce thin films with small grain size. Nevertheless, in some cases the films have poor compactness and many cracks [62]. In this context, adding a complexing agent and/or stabilizer to the precursor solution is one of the possible approaches to improve the morphology of the suggested p-MOX thin film [63,64,65]. However, the decomposition of volatile compounds and complexing agents in the prepared thin films at the annealing stage still may affect their quality [59]. Hence, identifying the evaporation temperature of the used solvent and the complexing agents in the precursor is very important. Generally, the sol–gel process can be summarized in five key steps, as follows [56]:

1. Synthesis of the ‘sol’ from hydrolysis and partial condensation of alkoxides.
2. Formation of the ‘gel’ via polycondensation to form metal–oxo–metal or metal–hydroxy–metal bonds.
3. Syneresis or ‘aging’, where condensation continues within the gel network, often shrinking it and resulting in expulsion of solvent.
4. Drying the gel either to form a dense ‘xerogel’ via collapse of the porous network or an aerogel, for example, through supercritical drying.
5. Removal of surface M–OH groups through calcination at high temperatures, up to 800 °C (if required).

Liquid-phase deposition processes are versatile methods to produce homogeneous coatings by spreading a solution onto a substrate and evaporating volatile compounds (solvent). In this context, sol–gel solution can be deposited using two methods: spin coating and dip coating.

- Spin Coating

**Figure 9:**
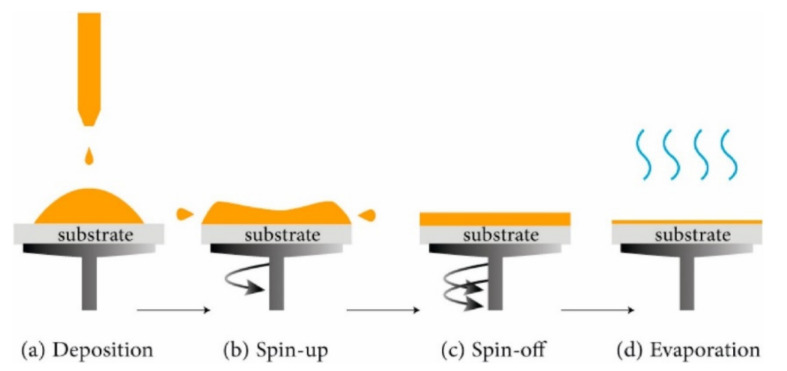
The four basic stages of spin coating: (**a**) deposition, (**b**) spin-up, (**c**) spin-off, and (**d**) evaporation. Reprinted with permission from [66].

Usually, the spin-coating technique embodies four stages, as shown in Figure 9 [66]. The required film can be achieved by layer-by-layer spin coating, followed by drying at a suitable temperature at each step. The fabricated films are heated in an ambient atmosphere for a few seconds to evaporate the solvent and finally annealed at high temperature to form the film. The spin-coating method has several advantages: thin and uniform coating, easy and quick depositing, reduced waste of materials, and low-cost techniques. However, the process lacks material efficiency, and spinning at high speeds is more difficult for large substrates.

Accordingly, the most significant parameter of spin coating is the precursor concentration. This significantly changes the overall morphology, structure, and performance of the proposed thin films. For instance, it was found that the crystalline quality and the average size of nanograins (from ~20 to ~36 nm) of NiO thin films increased when the precursor molarity was increased from 0.1 to 0.9 M [67].

**Figure 10:**
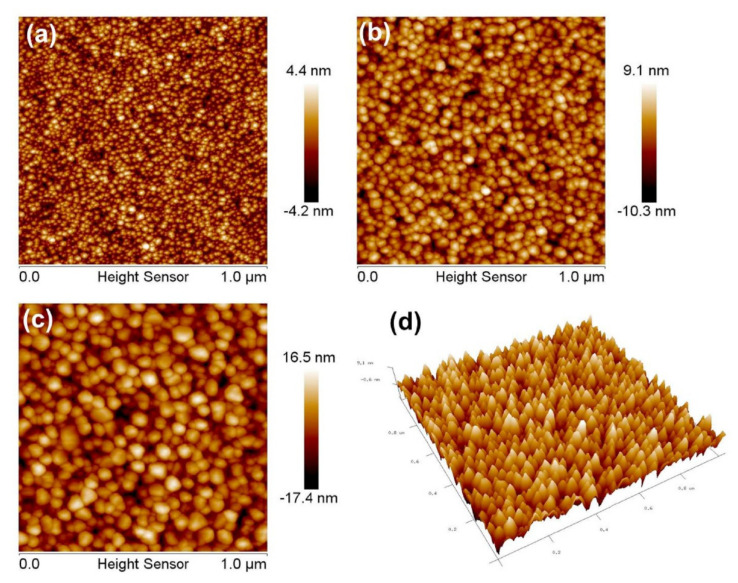
Surface morphology of NiO thin films dried at 250 °C and annealed at: (**a**) 400 °C, (**b**) 500 °C, and (**c**) 600 °C. (**d**) 3− D image of film annealed at 500 °C. Reprinted with permission from [68].

The thermal annealing effect also has an important influence on thin-film characteristics such as morphology, crystallinity, oxygen defects, etc., and is followed by a profound effect on optical and electrical performance. For instance, a higher drying temperature and higher annealing temperature help to grow uniform and relatively larger grains in NiO thin films (Figure 10) [68]. Additionally, annealing thin films at higher temperatures at partial Ar pressure increases grain growth due to increased adatom mobility. Furthermore, this also facilitates the removal of residual volatile components in the films, resulting in the formation of a monoclinic CuO structure [64]. Similarly, an enhancement of the crystallite size of CuO [69], NiO, and Co_3_O_4_ with annealing temperature was reported in [70]. The other important effect of the drying and annealing temperature is the formation of defects or vacancies in the material. For instance, NiO dried at 250 °C and annealed at 500 °C showed enhancement in the one-phonon, first-order longitudinal-optical (1P) LO peak (558 cm^−1^) over that of the two-phonon, second-order longitudinal-optical mode (2P) 2LO (1100 cm^−1^, compared to the sample dried at 160 °C and 200 °C). This enhancement indicates that NiO defect states became more occupied compared to the case of the samples dried and annealed at lower temperatures. Consequently, a decrement in the oxygen-vacancy concentration in the Co_3_O_4_ thin films was reported from 30.58% to 24.87% with an increase in the annealing temperature from 600 °C to 700 °C. [70]. Concerning annealing, another important parameter is the annealing duration, which is responsible for variation in the crystal structure. In this case, the grain size of the CuO decreased with increasing annealing duration [71]. This is mainly because variation in the preferred orientation due to the rearrangement of grain orientation was inevitable, resulting in the splitting of some grains. Subsequently, a reduction in grain size results in a reduction in carrier mobility [66].

Spin-coating speed is another key parameter that controls morphology, structural behavior, and optoelectrical properties of spin-coated films. For instance, crystallite size of CuO thin films was found to increase as spin speed increased to 3000 rpm [72]. Accordingly, the Eg value can be altered with higher spin speed due to the variation in the grain size and lower film thickness [66].

- Dip Coating

Dip coating, the deposition of a wet liquid film by vertical withdrawal of a substrate from a liquid coating bath at a constant speed, offers good control of the thickness [73]. Thereby, contrary to spin coating, the dip-coating deposition process is popular for thin films deposited on large and irregularly shaped surfaces. However, this technique is not widespread in the semiconductor industry due to the amount of precursor solution initially required in the reservoir [59]. Generally, the dip-coating process of film formation implies several technical stages [74]:

1. Immersion: the substrate is slowly dipped in the material precursor solution at a uniform speed.
2. Pull-up: the substrate is kept inside the solution for a fixed short duration, and then slowly pulled up.
3. Deposition: uniform deposition of thin layers on the substrate happens during the slow and steady pull-up stage. The withdrawal rate controls the layer thickness (faster pull-up results in thick layers).
4. Drainage: excessive liquid contents are simultaneously drained from the substrate, beginning during the pull-up stage and continuing outside the solution.
5. Evaporation: evaporation of solvent and formation of a thin layer happens. If the solvent is volatile (e.g., alcohol), evaporation begins during the pull-up stage and continues during the drainage sage.

**Figure 11:**
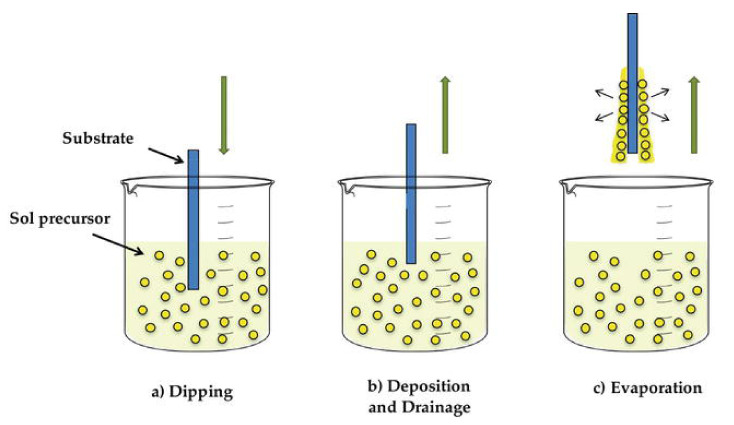
Schematic of the processes controlling thin-film formation in fast- and slow-rate deposition. Reprinted with permission from [75].

The experimental procedure for a standard dip-coating technique is shown in Figure 11 [75]. The dip-coating method has been used to prepare many p-type MOX thin films, such as CuO, NiO, Co_3_O_4_, and Cr_2_O_4_.

Like for spin coating, the pH value of the precursor is a significant feature, which controls the overall quality of the suggested film. Co_3_O_4_ thin films prepared with a pH value of 5 in precursor showed lower particle size (<10 nm) compared to a sample prepared with a pH value of 7, which was ascribed to hindering of the reaction mechanism by citric acid [76]. The molar ratio of the ingredients is another key factor in the dip-coating method. In [65], NiC_4_H_6_O_4_·_4_H_2_O, NH_3_·H_2_O, and C_3_H_8_O were maintained in the precursor with a ratio of 0.05:0.05:1 to grow NiO thin films with a cubic crystal structure, uniform and porous surface morphology, and consisting of nanocrystalline particles (diameters 20–30 nm). As mentioned above, withdrawal speed also highly affects the morphology and quality of the proposed films. Shariffudin et al. showed an increase in thickness and larger grain size of the CuO thin films when the withdrawal speed was reduced [77]. Additionally, dip cycles play an important role in controlling the thickness and morphology of p-type MOX thin films by enhancing the crystallinity and uniform distribution of nanoclusters over the substrate. Recently, Das et al. demonstrated a variation of gap energy (Eg), resistivity, mobility, and carrier concentration as a function of dip-coating cycles, in which 100 cycles demonstrated the highest Eg of 1.67 eV, 4.63 × 10^13^ cm^−3^, 4.53 cm^2^V^−1^s^−1^ together with a lower resistivity of 2.98 × 10^2^ Ω cm in CuO films [78]. On the other hand, the annealing temperature is a key parameter that controls the crystallography structure. For instance, Ray et al. showed a change in the crystal phase (Cu_2_O to CuO) according to annealing temperature (>400 °C) [79].

#### 3.2.2. Spray Pyrolysis

**Figure 12:**
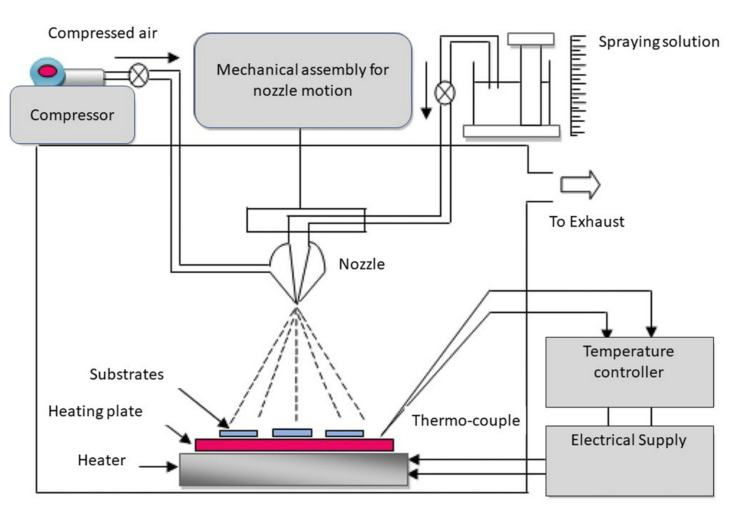
Schematic of spray-pyrolysis technique. Reprinted with permission from [81].

Usually, the spray pyrolysis method involves spraying a solution on top of a heated substrate, causing a chemical-compound layer to be formed due to the reactions of the constituents [80]. Typical spray-pyrolysis equipment consists of an atomizer, precursor solution, a substrate heater, and a temperature controller [68]. A schematic of the spray-pyrolysis process is shown in Figure 12 [81]. In this process, the solution is atomized in small drops (aerosol). The generated solution droplets are then very rapidly heated at a given temperature, thus passing through several stages: (1) evaporation of the solvent from the surface of the droplets; (2) drying the droplets containing the precipitated solute; (3) annealing of the precipitate at high temperatures (thermolysis); (4) formation of microporous particles of defined phase composition; (5) formation of solid particles; and (6) sintering of solid particles [82] Among different physical and chemical deposition techniques, the spray pyrolysis method offers better control over the preparation parameters to fabricate devices based thin films for numerous emerging applications. Consequently, several p-type MOX thin films, such as CuO, NiO, Cr_2_O_3_, and Co_3_O_4_ have been prepared by spray pyrolysis [83,84,85,86].

Usually, a longer spraying duration increases the grain size and enhanced surface-layer coverage. For instance, higher crystallization (crystallite size of 24–31 nm) was achieved in an NiO sample with a higher thickness (505 nm). This was due to the growth of grains by increasing the thickness as a function of spraying time [84]. Accordingly, thicker films exhibit a higher scattering of incident light, leading to decreased transmittance in NiO thin films [87]. Additionally, Daira et al. showed that the spray number influences the transmittance spectra of CuO thin films. Indeed, transmittance in the range 400–800 nm was found to decrease from 35 to 5% as a function of the spray number due to the high electronic transitions between the valance and conduction band [88]. Additionally, Eg of CuO thin films was found to decrease from 2.14 to 1.85 eV as a result of increasing crystallite size from 4 to 5.7 nm when increasing the spraying number (from 25 to 125) [83].

The substrate temperature is another significant parameter that controls the surface morphology of thin films. For instance, it was shown that the surface of CuO thin films sprayed at 300 °C was not fully covered due to insufficient heating for the complete decomposition of CuO [83]. However, increasing the substrate temperature up to 350 °C films resulted in compact and uniform growth of a CuO thin film over the substrate. Meanwhile, CuO films deposited at 400 °C uniformly covered an FTO-coated glass substrate with an interconnected nanoparticle-like (size of nanoparticles, ~150–200 nm) network. Typically, variation in crystallinity alters the optical properties of the material. In addition, the morphology and crystallinity of the material can be altered by varying the substrate temperature and annealing treatment. The substrate temperature affects the nucleation and growth of crystalline thin films, and annealing greatly enhances crystallinity. Increasing the substrate temperature was found to increase the crystallinity of NiO due to the variation of the defects and strains [89]. For instance, NiO thin films prepared at higher substrate temperatures show higher Eg due to adatom mobility, which also enhances crystallite size and crystallinity [66]. Crystallinity can be increased in Co_3_O_4_ by annealing at higher temperatures (>700 °C) [90]. Furthermore, in another study, the structural quality of Co_3_O_4_ was improved, as well as the porosity, by increasing the annealing treatment from 300 °C to 500 °C [85].

In conclusion, spray pyrolysis is a very valuable, cost-effective deposition process that employs simple equipment. Thin films produced with this process have a large surface area, are homogeneous, and have a potential for mass production. However, spray pyrolysis still has some disadvantages, such poor crystal quality of films, thermal decomposition, and vapor convection. Vapors are generated due to temperature differences, which restricts the source from binding with the substrate.

#### 3.2.3. Electrodeposition

**Figure 13:**
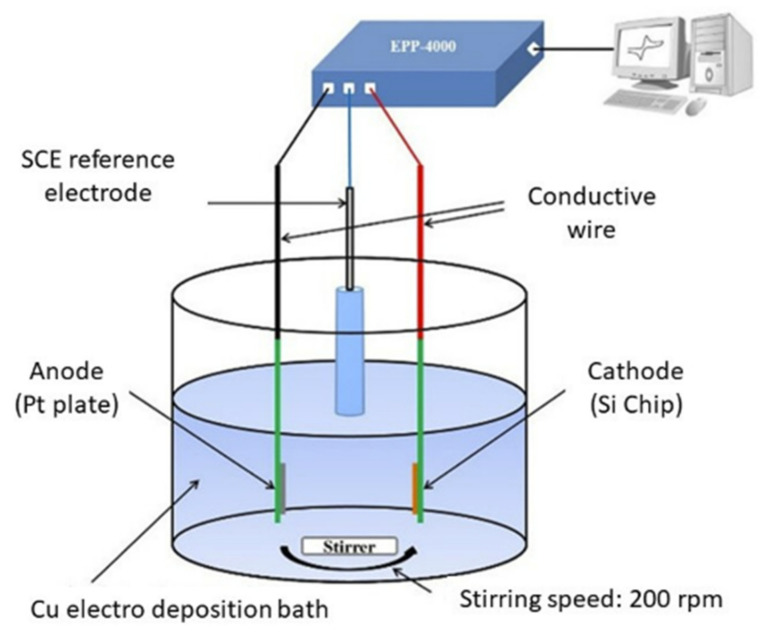
Three-electrode system plating cell. Reprinted with permission from [91].

The electrodeposition method is one of the standard methods of electroplating. It consists of an electrolyte containing the appropriate ions and three electrodes, including a working electrode, a counter electrode, and a reference electrode, as shown in Figure 13 [91]. The electrodeposition of thin films is a viable alternative to vacuum-based deposition processes, such as sputtering, plasma deposition, or chemical vapor deposition. It has significant advantages, such as growth at room temperature.

**Figure 14:**
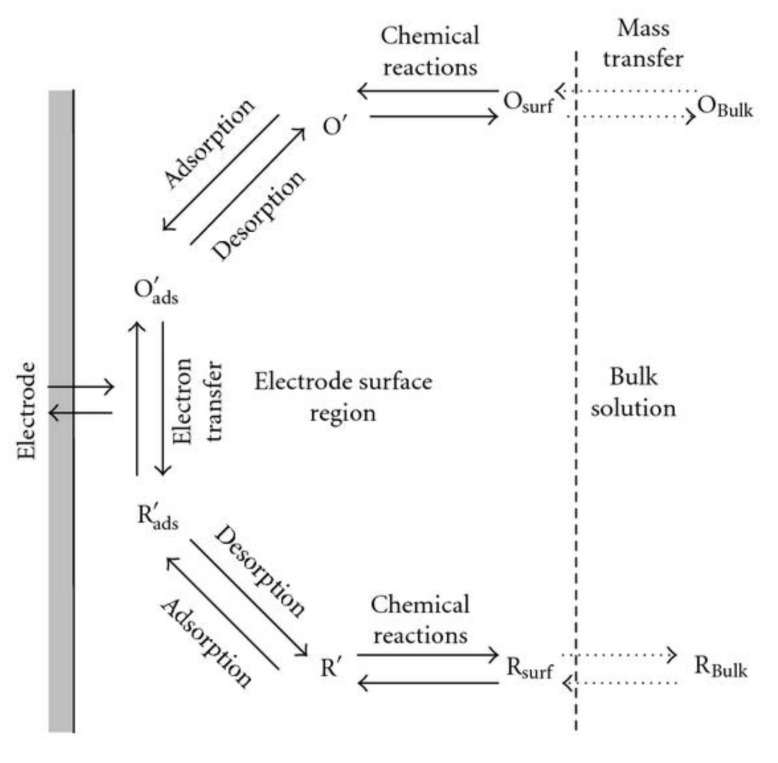
Pathway of a general electrode reaction. Reprinted with permission from [92].

Usually, a small DC power, typically in the range of milliwatts, is applied across the anode and the cathode. The technology of electrochemical deposition of metals and alloys involves the reduction of ions from aqueous, organic, and fused salt electrolysis. The deposition of material species involves the reduction of ions in the solution, such as $M_{Sol}^{Z+}$ + Ze → $M_{lattice}$. The seemingly simple single reaction requires pre- and post-complex steps before contributing to the whole deposition process, as depicted in Figure 14 [92]. This is a reaction of charged particles at the interface between a solid metal and a liquid solution. The two types of charged particles, ions and electrons, can cross the interface. Hence, four types of fundamental areas are involved in the due process of deposition: (1) electrode–solution interface as the locus of the deposition process; (2) kinetics and mechanisms of the deposition process; (3) nucleation and growth processes of the deposits; and (4) structure and properties of the deposits. Mallik et al. provide an explanation of each consecutive section in [91].

**Figure 15:**
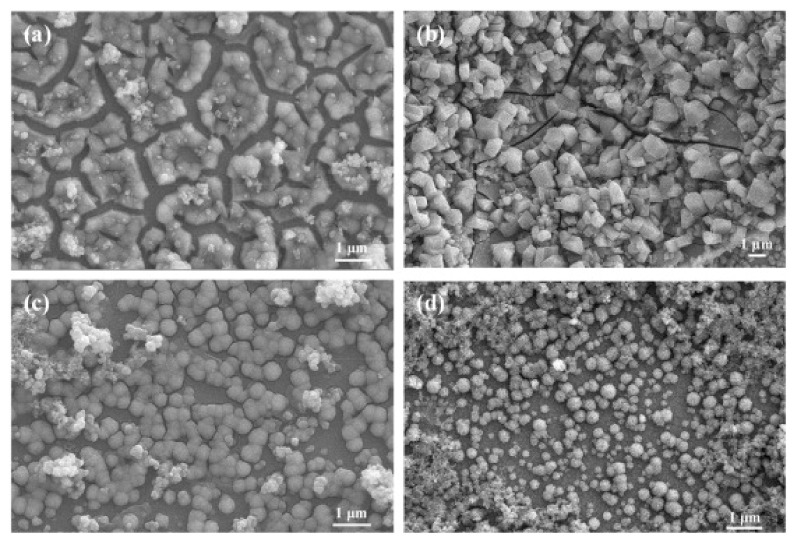
Images of fabricated Al:NiO thin films at different current densities. (**a**) (4 mAcm^−2^), (**b**) (5 mAcm^−2^), (**c**) (6 mAcm^−2^), and (**d**) (7 mAcm^−2^). Reprinted with permission from [93].

Usually, electrodeposition temperature, pH value of the electrolyte, deposition voltage/current, and annealing temperature significantly control the properties of the produced thin films. Firat et al. showed variation in surface morphology as a function of applied current density in the galvanostatic electrodeposition of Al:NiO thin film [93]. In this study, the surface morphology turned from rectangular to spherical formations as the current density was altered from 4 mAcm^−2^ to 7 mAcm^−2^ (Figure 15) [93]. Wu et al. also employed two potentiostatic conditions of 0.9 V/Ag/AgCl and 1.05 V/Ag/AgCl to demonstrate the dependence of pore size on applied potential, in which lower potential led to larger pore size [94]. Furthermore, as opposed to applying constant voltage, a pulse voltage is also helpful to create porous and rough NiO thin films in potentiostatic electrodeposition [95]. On the other hand, different surfactants can be added to the electrodeposition electrolyte to alter its surface morphology. When an anionic surfactant, such as sodium dodecyl sulfate, is added to the electrolyte, the hydrophilic surface of NiO thin film becomes super hydrophilic, resulting in smaller pores than in film deposited without the surfactant [96,97]. Additionally, Ortiz et al. showed the importance of using surfactant (glycine) in the electrolyte to grow NiO with a smaller grain size [98]. Subsequently, a relatively low current density (−0.05 mA/cm) was also used to achieve higher surface area, which was ascribed to smaller grain size, higher porosity, and homogeneity of NiO film [99].

Generally, the annealing temperature controls the structural properties, including phase transition, and hence the optical and electrical properties. For instance, annealing n-Cu_2_O at 500 °C for 30 min in air has been used to transfer n-Cu_2_O into single-phase p-CuO [100,101]. After annealing at 500 °C, a highly porous Co_3_O_4_ thin film was grown for integration into supercapacitors [102]. Moreover, the morphology of MOX thin films prepared by electrodeposition can also be tuned with annealing treatment. In this context, it was discovered that Co_3_O_4_ nanosheets transform into nanoparticles upon heating at 300 °C or higher [103]. On the other hand, composite structures such as Ni–Cr_2_O_3_ [104] and Cr_2_O_3_/Al_2_O_3_ [105] were also prepared by electrodeposition.

## 4. Sensing Properties of P-Type MOX Thin Films

### 4.1. Reducing Gases

#### 4.1.1. Volatile Organic Compounds (VOCs)

VOCs are chemical compounds containing at least one carbon atom and one hydrogen atom in their molecular structure [106]. In this review, the detection of some common VOCs, including acetone (C_3_H_6_O), ethanol (C_2_H_5_OH), propane (C_3_H_8_), benzene (C_6_H_6_), formaldehyde (HCHO), n-butanol (C_4_H_9_OH), and methanol (CH_3_OH), will be investigated. We will, however, demonstrate that p-type MOX sensors can be applied to multiple applications through their sensing efficiency in detecting VOCs. Chemicals such as VOCs are found in our daily lives. As these compounds are emitted in industrial estates, plants, transport, agriculture, and waste combustion, VOC detection has a variety of applications, such as ensuring human and animal health, protecting the environment, and controlling food quality [107]. Moreover, VOCs can be generated internally within a person’s body, which are called endogenous VOCs, or outside the body through food consumption or environmental exposure, which are called exogenous VOCs [108]. Nowadays, VOCs are used as biomarkers for disease identification through breath analysis. Therefore controlling VOCs in the human body is an advanced medical application.

The detection of acetone has been investigated using many p-type MOX thin films, such as NiO, CuO, Cr_2_O_3_, and Co_3_O_4_. Moreover, doped structure, heterojunction, and functionalized p-type MOX thin films have been studied. When studying thin films, it is essential to examine the effect of the film’s thickness on its sensing performance. NiO thin films grown by magnetron sputtering showed good response to 3 ppm of acetone at high temperatures [109]. In the study, the thicknesses of the layers were examined to determine their effect on acetone response. Specifically, the authors found that the response decreased (small variation) from 6.6% to 6.3% towards 17.5 ppm of acetone when the thickness increased from 25 nm (grains with an average size of about 10 nm) to 50 nm (grains with an average size of about 15 nm) [109]. However, these results may be due to increased porosity and enhanced surface-to-volume ratio as the grain size is reduced. However, the correlation between sensing performance (response and sensors kinetics) and film thickness is still unclear based on the results of several works that reported on p-type MOX [110]. The general reaction that occurs between acetone and the surface of MOX can be described by Equation (4).

(4) $$C_{3}H_{5}OH+8O_{\left(ads\right)}^{-}\to 3CO_{2}+3H_{2}O+8e^{-}$$

The sensing performance is influenced by different factors, such as particle size, defect, active sites, etc. [111,112]. Sai et al. studied thickness-related effects on NiO’s response to VOCs [110]. The thicker the film, the higher and faster the response. According to the authors, thick NiO films have greater porosity because of their open microstructure with the presence of macropores compared to thinner films with compact microstructures. The authors attributed these results to the high porosity, high specific area, and high active sites, which increased the absorption rate and caused the high response. On the other hand, NiO thin film with 50 nm thickness was found to show superior sensing characteristics (low response, recovery times, and detection limit (LOD)) in comparison to film with a 100 nm thickness. Note that LOD is defined as the lowest concentration of the chemical compound (gas) that can be reliably detected. The LOD for a sensor with a 50 nm thick film was 0.1 toward 5 ppm of ethanol and 1 toward 50 ppm of acetone. For a sensor of 100 nm, the LOD was 0.4 and 8 for a given concentration [111]. The general reaction that occurs between ethanol and the surface of MOX can be described by Equation (5). Interestingly, in another work, thickness had no direct impact on CuO sensors towards acetone [112]. Among samples with thicknesses of 120, 192, 240, and 288 nm, the highest response was found for the sample of 240 nm thickness. The response was 380 toward 300 ppm at 300 °C, with long-term stability (180 days). Furthermore, the sensors remained responsive even at high humidity levels (90%). The thickness effect on acetone detection has been investigated for other p-type MOX, such as Co_3_O_4_ [113].

(5) $$C_{2}H_{5}OH+6O_{\left(ads\right)}^{-}\to 2CO_{2}+3H_{2}O+6e^{-}\;$$

**Figure 16:**
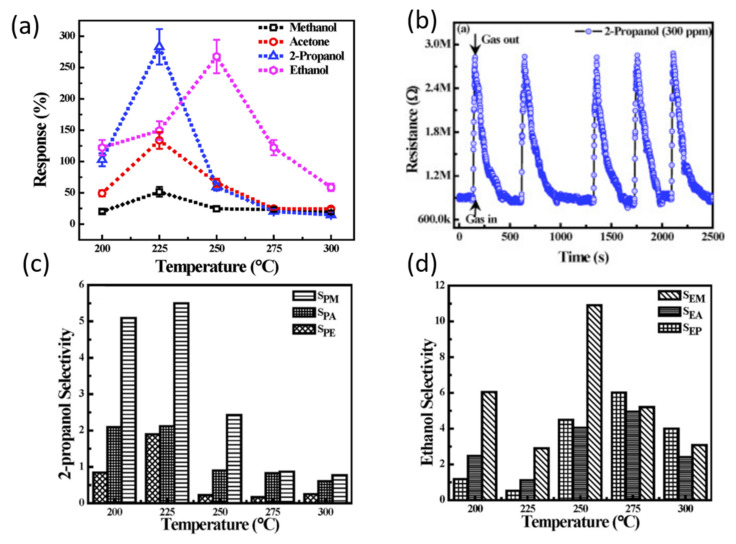
The sensing performance of CuO thin films. (**a**) The response, ($\frac{R_{gas-}R_{air}}{R_{air}}$), of CuO towards 300 ppm of several VOCs at different temperatures, where $R_{gas}$ and $R_{air}$ are the resistances measured in presence of gas and air, respectively. (**b**) The repeatability of CuO sensor towards 300 ppm of 2 propanol at 300 ℃. (**c**,**d**) the selectivity study of 2 propanol and ethanol over other VOCs. Subscripts P, M, A, and E refer to propanol, methanol, acetone, and ethanol, respectively. Reprinted with permission from [114].

Sensor characteristics are dependent on several factors related to gas testing, such as working temperature, humidity, gas concentration, etc. A CuO thin-film sensor was developed to detect a variety of VOCs, such as 2-propanol, ethanol, acetone, and methanol [114]. In this study, the response of the CuO sensor was shown to be strongly related to temperature. Tests were conducted at temperatures ranging from 200 °C to 300 °C. Figure 16 illustrates the response and selectivity of CuO at different temperatures for various chemical compounds. The optimal temperature for the detection of 2-propanol, methanol, and acetone was 225 °C, whereas 250 °C was optimal for the detection of ethanol. The response of CuO towards 300 ppm of ethanol was ~260% at 250 °C, while the response towards 300 ppm of 2-propanol was ~280% at 225 °C. Additionally, the selectivity of ethanol and 2-propanol against VOCs was investigated. A cross sensitivity was observed, and the sensor did not display full selectivity towards a single VOC.

P-type MOX are capable of detecting VOCs at RT. In this context, Co_3_O_4_ was investigated as an appropriate thin-film material for acetone detection at RT [113]. A response of 235 towards 50 ppm was obtained at RT. The sensor showed an excellent detection limit of 1 ppm and had an ultra-fast response time of 6 s and a recovery time of 4 s at a high humidity level (up to 90%). Moreover, the sensor showed perfect repeatability and excellent selectivity towards acetone over some biomarkers, such as ethanol (C_2_H_5_OH), xylene (C_8_H_10_), toluene (C_7_H_8_), ammonia (NH_3_), and acetaldehyde (C_2_H_4_O). Low detection limits of acetone (below 2 ppm) with fast sensor kinetics at high humidity levels make Co_3_O_4_ an attractive material for breath-analysis applications for diabetes diagnosis. Moreover, acetone detection at RT offers a potential advantage for gas sensors with low power consumption.

Pristine MOX thin films in general, comprising p-type, have issues with selectivity, stability, and power consumption. Numerous approaches may be applied to overcome these issues, including bulk doping, surface functionalization, and heterostructures.

Bulk doping can increase the response, enhance selectivity, and improve long-term stability [115,116,117]. It was shown that doping CuO thin film with Cr increases response and selectivity toward 3.2 ppm of acetone when operating at 450 °C at a relative humidity of 50%. The response increased from 2 to 3.2 with the addition of Cr. The detection limit was found to be 0.4 ppm, and the selectivity towards acetone was verified over other compounds, such propane and ethylbenzene [115]. In another work, doping CuO thin films with Au, Cr, Pd, Pt, and Sb resulted in an improved response towards propane (1ppm) at 250 °C. CuO doped with Cr, on the other hand, appears to enhance long-term stability because the dopant stabilizes the grains and increases porosity. Moreover, the improved response may be attributed to the electronic sensitization induced by substituting Cr^3+^ ions into CuO lattice. In this context, the hole density is reduced by generating the donor state due to the substitution effect, which decreases the material conductance and affects the response [115]. Moderation of hole density was observed in an ethanol sensor based on Li(I)-doped CuO thin film. It was reported that Li(I)-doped CuO showed enhanced ethanol resistance at 300 °C when exposed to 1000 ppm. In this study, the authors attributed the improved performance to the elimination of point defects, such as Cu vacancies, due to the incorporation of Li(I) into the CuO lattice, in conjunction with tuning the hole density [116,117]. Moreover, NiO thin film doped with Al was used to detect methane (NH_4_) at room temperature [118]. The sensor exposed towards 5 ppm of NH_4_ showed good repeatability (2 cycles), and the response, response time, and recovery times were 58%, 1373 s, and 95 s, respectively.

In addition to using pristine p-type MOX, composite structures, such as a combination different MOX, are also used to detect VOCs, for example, CuO/SnO_2_ for triethylamine detection [119]; CuO/Ga_2_O_3_, [120] CuO/WO_3_, ZnO/NiO, and Co_3_O_4_/ZnO for acetone detection [121,122,123]; TeO_2_/Co_3_O_4_, Fe_2_O_3_/Co_3_O_4_, ZnO/Co_3_O_4_, and SnO_2_/NiO for ethanol detection [124,125,126,127]; PtO_2_/CuO for butanol detection [128]; and (CuO-Cu_2_O)/ZnO:Al for VOC detection (specifically butanol) [129].

Jiabin et al. investigated an NiO/SnO_2_ thin film sensor towards ethanol [130]. Thanks to the p-n junction formed at the interface between two MOX, the achieved sensor was capable of better sensing performances than the pristine sensor. At 250 °C, the response was 7.9 towards 100. However, the formation of heterostructures enables the sensors to be sensitive to very low ethanol concentrations (100 ppb). Another work in which low concentrations of acetone (250 ppb (0.25 ppm)) were detected with CuO/Ga_2_O_3_ was reported by Katarzyna et al. [120]. Compared with pristine CuO, the sensors based on CuO/Ga_2_O_3_ heterostructure demonstrated improved sensing properties. Decreases in response time (from 431 s to 394 s) and recovery time (from 862 s to 563 s) were observed with high stability at high humidity levels, although the response did not increase significantly. n-ZnO-p-NiO heterostructures demonstrated ultra-fast response times (13 s and 18 s, respectively) and strong responses to acetone concentrations ranging from 1 ppm to 500 ppm at 300 °C [122].

Briefly, the improved sensing performance was ascribed to the formation of a pn junction at the interface. First, oxygen adsorption creates a space charge region in both n- and p-type MOX materials. At the interface, electrons move from the MOX with a higher energy state to the MOX with a lower energy state until Fermi levels are equilibrated. As a result, a larger space-charge region than that formed during oxygen adsorption may be formed. When the n-MOX–p-MOX heterostructure sensor is exposed to VOCs, the pre-adsorbed oxygen reacts with VOC molecules, transferring electrons back to the n-MOX–p-MOX system. The electrons combine with the holes of p-MOX, resulting in a decreased hole density in the valence band and an increased electron density in the conduction band of n-MOX. This results in an overall change of sensor conductance, as well as the gas response [131].

On the other hand, the detection of VOCs can also be improved through the functionalization of MOX thin films by other metal nanoparticles. Noble metals are typically utilized to further enhance the sensing response through two different mechanisms: the spillover effect or electronic sensitization. In this context, CuO/Cu_2_O functionalized by Ag displayed improved response (2.9 to 7.7 times higher) for various VOCs, including toluene, ammonia, ethanol, propanol, and acetone. The high response is due to the chemical and electronic sensitization induced by Ag nanoparticles. The chemical sensitization of NiO thin films decorated with Pd was investigated in [132]. A Pd/NiO sensor demonstrated a good response (10.1) when operated at 250 °C towards 16 ppb of formaldehyde. Pd nanoparticles contributed to oxygen dissociation during oxygen adsorption through the spillover effect, affecting the space-charge layers, increasing the interaction surface and the formaldehyde-adsorption rate.

The sensing results reported on p-type MOX towards VOCs are summarized in Table 1.

#### 4.1.2. Hydrogen (H_2_)

Hydrogen is a flammable and explosive gas (when its concentration exceeds 4%) with a high deflagration index [133]. Therefore, the detection of H_2_ is essential to ensure its safe utilization, especially in energy-based applications, such as fuel-cell technologies. Many hydrogen sensors have been produced using different p-type MOX. CuO and NiO have been extensively studied, while few studies have been conducted on pristine Co_3_O_4_ and Cr_2_O_4_. However, the latter are usually used as additives or sensitizers for H_2_ detection due to their high catalytic properties. NiO thin films were grown using magnetron sputtering, and capability of detecting hydrogen was studied by Chou et al. [134]. The authors displayed an LOD (<50 ppm) and fast response (7 s) towards H_2_ concentrations ranging from 50 ppm to 1% ppm at temperatures below 350 °C [134]. Guziewicz et al. examined NiO thin film grown by magnetron sputtering for the detection of low H_2_ concentrations in air at operating temperatures between 130 °C and 145 °C [135]. In this study, the authors also examined the effect of the thickness of NiO thin films on hydrogen detection, similar to what was discussed in the VOC section. More details can be found in [135]. In other work, Zahira et al. optimized NiO thin films using the Taguchi design for hydrogen detection [136]. To obtain a high degree of NiO crystal orientation or the highest texture coefficient, several factors were considered, such as the precursor concentration, annealing time, and annealing temperature. NiO films with a nickel-chloride dehydrate precursor concentration of 0.08 M, annealed at 380 °C for 3 min, exhibited the highest texture coefficient. NiO samples were then exposed to 100 ppm of hydrogen at 300 °C, and the response was 55. The high response was probably due to the strong texturing of crystallites of NiO. Thin films of Cr_2_O_3_ were produced by magnetron sputtering, and their suitability for detecting H_2_ was demonstrated; however, the Cr_2_O_3_ sensor suffered from a selectivity issue [137]. The grain size was controlled by thermal annealing at a temperature ranging from 350 °C to 450 °C. Sensing characteristics, such as response and selectivity, can be tailored by annealing thin films. Indeed, here, the size has no direct effect on improving gas detection; the response is affected by many factors, such as operating temperature, humidity, hole concentration, grain boundary, and the metal vacancies in p-type MOX. In addition, a low-power sensor for the detection of hydrogen has been reported but not extensively. Using thermal oxidation, a highly sensitive hydrogen sensor based on NiO thin films was developed [138]. A high response (310%) was obtained towards 1000 ppm at RT, with a very fast response (6 s) and rapid recovery (0.5 s). On the other hand, detection at room temperature has limitations because of the high detection limits achieved. The general reaction that occurs between H_2_ and the surface of MOX can be described by Equation (6).

(6) $$H_{2}+O_{\left(ads\right)}^{-}\to H_{2}O+e^{-}\;$$

On the other hand, combining different MOX can lead to an enhancement of H_2_ detection. In this context, many heterostructures have been used to detect H_2_ and enhance the sensing characteristics, with composite structures such as Co_3_O_4_/WO_3_ [139], Co_3_O_4_/SnO_2_ [140], CuO/TiO_2_ [141], CuO/Al_2_O_3_ [142], NiO/TiO_2_ [143], CuO/Al_2_O_3_ [142], Pd/TiO_2_/CuO [144], NiO/Au [145], and NiO/Pd [146].

**Figure 17:**
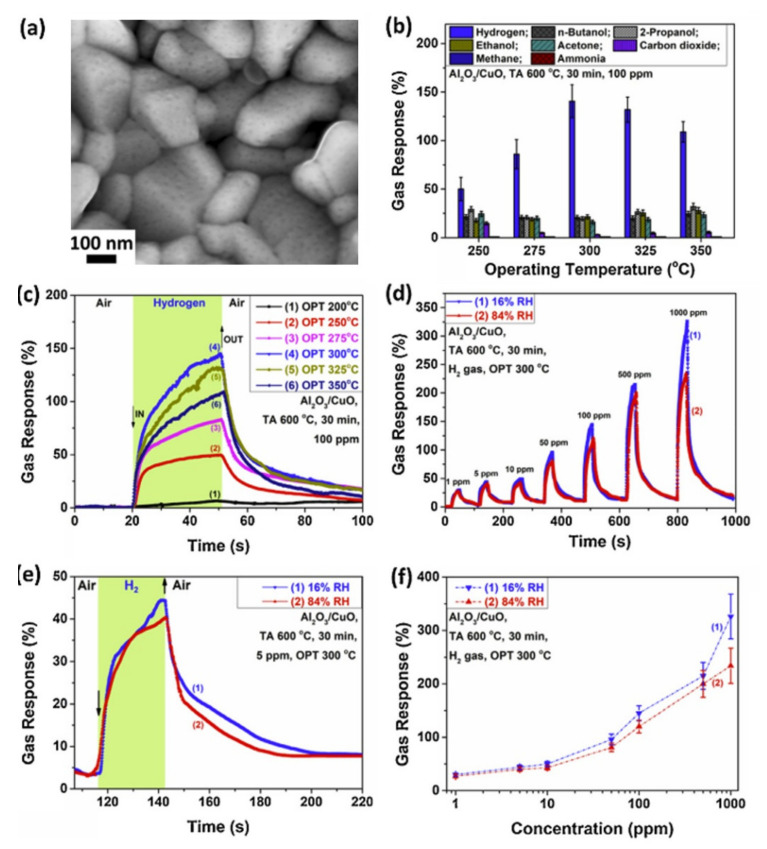
(**a**) SEM image of CuO/Al_2_O_3_ heterostructure with thermal annealing (TA) at 600 ℃ for 30 min. (**b**) Responses towards different gases have the same concentration (100 ppm) versus different operation temperatures (OPT). (**c**) Dynamic response of CuO/Al_2_O_3_ heterostructure at different working temperatures. (**d**) Dynamic response of CuO/Al_2_O_3_ heterostructure towards various H_2_ concentrations ranging from 1 ppm to 1000 ppm at different humidity levels. (**e**) Dynamic response of CuO/Al_2_O_3_ heterostructure towards 5 ppm of H_2_ at different humidity levels. (**f**) Response (%) versus different concentrations of CuO/Al_2_O_3_ heterostructure at 300 ℃. Reprinted with permission from [142].

In p-type MOX thin films, selectivity is one of the most critical aspects. Compared to Co_3_O_4_, Co_3_O_4_/SnO_2_ heterojunction showed increased response, better stability, excellent reversibility, and enhanced selectivity towards 50 ppm at 300 ℃ of H_2_ over CO, H_2_S, and NH_3_ [140]. CuO/TiO_2_ led to an increase in the response from 1.3 to 2.55 towards 125 ppm at 300 ℃, as well as an increase in the electrical baseline stability compared with a pristine sensor [141]. The enhanced sensing performance was attributed to the space-charge region formed at the interface between two MOX, as explained previously for VOCs. It is well known that MOX sensors suffer from high humidity levels. It has been shown that composite structures can be used to achieve high stability at high humidity as well as good selectivity, such as by combining p-type MOX with dielectric metal oxide. Recently, Laupan et al., studied the CuO/Al_2_O_3_ heterostructure towards H_2_ at temperatures ranging from 275 to 350 °C [142]. The response was 80% towards 100 ppm when the sensors operated at 350 C, and the response and recovery times were 20.8 s and 59.9 s, respectively. In this work, a significant feature of the sensor was its high selectivity to H_2_ over butanol, isopropanol, acetone, ethanol, and ammonia in all temperature ranges. Furthermore, the sensor was humidity-independent, and it showed similar performance when the humidity changed from 11% to 79% (Figure 17). In addition, the sensors were able to react with H_2_ and return to their initial state within a few seconds. The CuO/Al_2_O_3_-based hydrogen sensor showed perfect characteristics for breath-analysis applications.

It has been shown that decorating NiO with Au increases the response and lowers the LOD [145]. After functionalization with Au, the LOD was found to be dramatically reduced from 123 ppm to 2.7 ppm when the sensor was operated at 125 °C. Together with Au, Pd was also reported as a good sensitizer for H_2_ detection [146,147]. Pd/CuO was reported to undergo chemical activation by Pd, where a high response and a high selectivity to H_2_ were achieved over CO and NH_3_ [148]. Enhanced sensing performances induced by decorating MOX thin films with metal nanoparticles can be explained by the spillover effect or chemical sensitization caused by metals such as Au and Pd nanoparticles. They act as a catalyst, accelerate gas dissociation at the surface, and enhance the adsorption rate of H_2_.

Sometimes, instead of using air, which is typically used to restore the baseline of the sensor, N_2_ is used. Testing sensors in an oxygen-free environment revealed that they were unresponsive compared to testing them in dry air [149]. This result shows the crucial role of oxygen in sensing mechanisms.

The sensing results reported on p-type MOX towards H_2_ are summarized in Table 1.

#### 4.1.3. Ammonia (NH_3_)

Ammonia is an extremely irritating, colorless gas with a suffocating smell. NH_3_ is a chemical compound commonly used in different industries, such as in agriculture for fertilizers. Inhaling a large amount of ammonia can cause immediate burning in the nose and throat, as well as respiratory problems, while low concentrations of ammonia can cause skin and eye irritation. NH_3_ from farms, industrial estates, and products such as cleaning products used in our daily lives is spreading. Therefore, safety precautions must be taken. This can be done with the use of highly performant chemical sensors.

In recent years, investigation on RT detection of ammonia has become increasingly popular. Detection at RT allows for easier development of portable gas sensors with no need for external heat. However, detection at room temperature suffers from two important limitations: low response and limitation of the limits of detection. The general reaction that occurs between NH_3_ and the surface of MOX at room temperature can be described by Equation (7) Accordingly, a recent study by Cynthia et al. reported an RT ammonia sensor using CuO/SnO_2_ composites [150]. The composite was deposited using magnetron sputtering and tested towards NH_3_ concentrations ranging from 10 to 150 ppm, showing good selectivity over ethyl ethanol (CH_3_CH_2_OH), dimethyl ketone (CH_3_COCH_3_), methylbenzene (C_6_H_5_CH_3_), and formaldehyde (HCHO). The sensor response increased with increased concentration, leading to a maximum response of 11.21 at 100 ppm, then decreasing as NH_3_ concentration increased to 150 ppm. Based on these findings, this study sheds light on another critical issue, namely ‘’sensor poisoning’’. This happens more frequently when operating the sensor at room temperature. When the surface of MOX is exposed to high concentrations of the target gas, the adsorbed molecules require kinetic energy, such as that provided by photons or increased temperature, to desorb from the surface. Otherwise, the sensor is less responsive and/or less stable. In another study, the same group (Cynthia et al.) enhanced the response and selectivity of CuO/SnO_2_ by sputtering ZnO to form a CuO/SnO_2_/ZnO composite. The good sensing performances may be ascribed to the higher surface interaction. The latter demonstrated long-term stability (6 months) [151].

(7) $$4NH_{3\left(ads\right)}+3O_{2}^{-}\to 2N_{2}+6H_{2}O+3e^{-}$$

On the other hand, a composite structure composed of p-type MOX and n-type transition-metal dichalcogenides (TMDC) has been suggested to enhance ammonia-sensing performances [152]. The sensing mechanism of the p-n heterojunction should, however, be the same as explained in the previous section. Due to the differences in Fermi levels between n- and p-type MOX, band bending occurs. Electrons and holes are transferred between them until an equilibrium in Fermi levels is achieved and a space-charge region is formed. This space-charge region is modulated by the presence of NH_3_. However, the final performance of the composite is influenced by the properties of the materials used to construct the composite, such as catalytic effects, defects, active sites, and adsorption energies. In this regard, DFT studies showed strong adsorption energy on the surface of MoS_2_ to NH_3_ over reducing gases such as CO and H_2_ [153]. In fact, this could provide the basis for improved ammonia detection, as reported by Sharma et al., who observed excellent improvement in response, short- and long-term stability, sensor kinetics, and selectivity at RT for n-MoS_2_/p-CuO [152].

**Figure 18:**
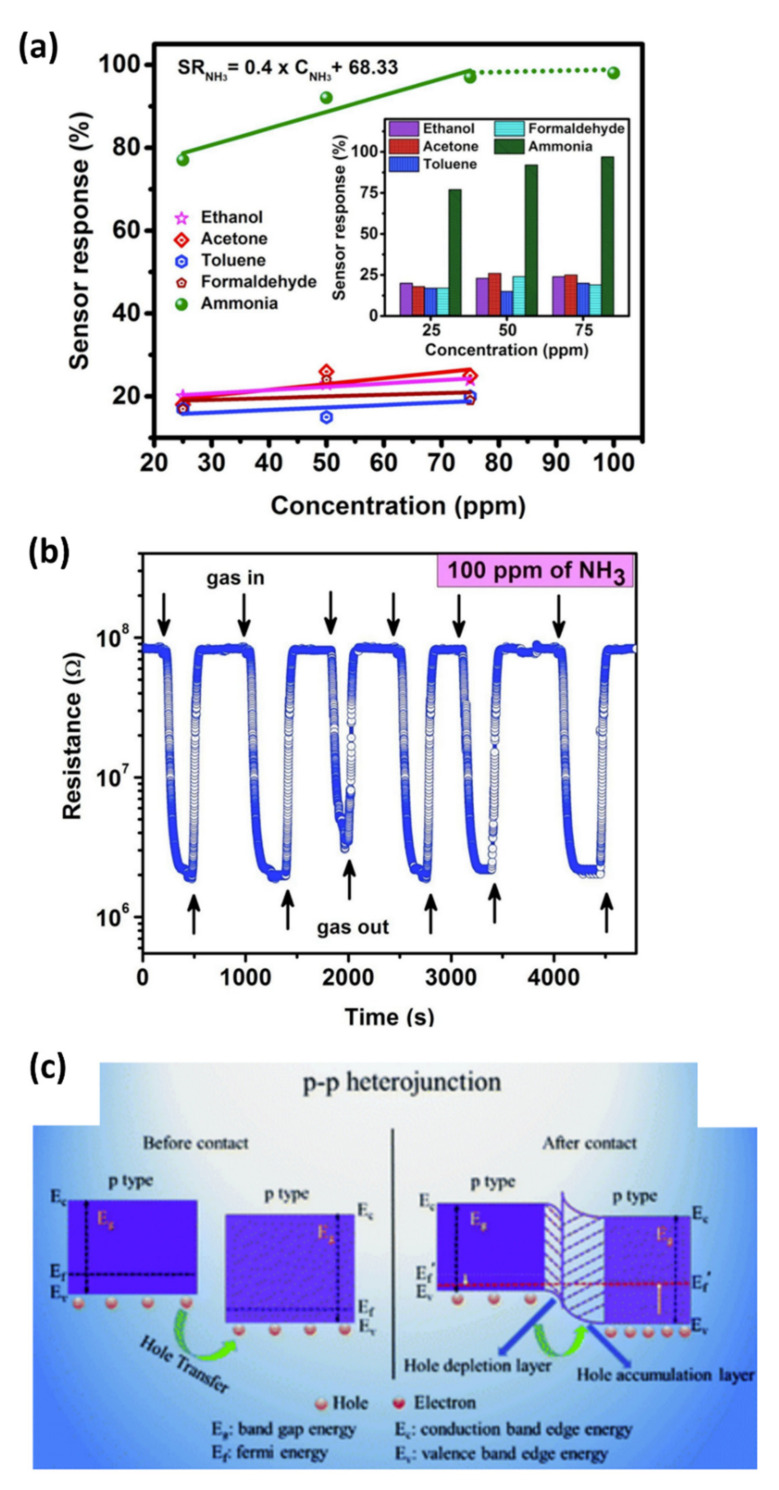
(**a**) Response and selectivity of Cr_2_O_3_/CuO thin films at RT towards many NH_3_ concentrations over several gases. (**b**) Repeatability of Cr_2_O_3_/CuO thin film for 100 ppm of NH_3_ at RT. Reprinted with permission from [154]. (**c**) Energetic-band diagram of pp heterojunction. Reprinted with permission from [155].

**Figure 19:**
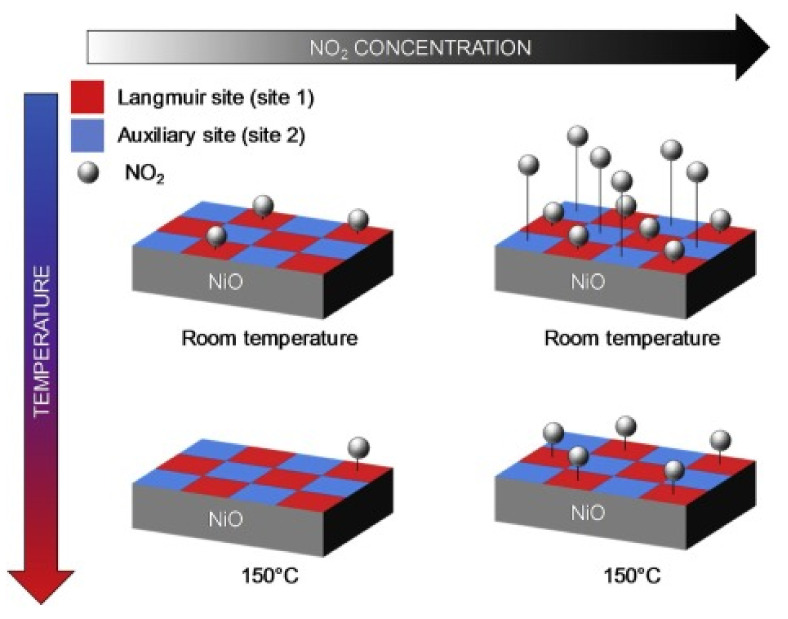
Schematic illustration of the proposed model for NO_2_ detection by the nanoporous NiO film as a function of temperature and NO_2_ concentration. Reprinted with permission from [163].

Besides p-n heterostructures, p-p heterostructures were demonstrated by combining two MOX with the same p-type conductivity to enhance NH_3_. Ponmudi et al. reported a p-Cr_2_O_3_/p-CuO composite prepared by magnetron sputtering as an RT ammonia sensor for the first time (Figure 18a,b) [154]. Despite a weak response of 77% towards 25 ppm, the sensor exhibited a relatively fast response time and recovery time of 11 s and 14 s, respectively. Additionally, Cr_2_O_3_/CuO composites were highly selective for NH_3_ over ethanol (C_2_H_5_OH), acetone (CH_3_COCH_3_), toluene (C_6_H_5_CH_3_), and formaldehyde (HCHO). However, this is explained by the pp junction formed at the interface between Cr_2_O_3_ and CuO. In general, since the majority of carriers are holes, the sensing results are due to the formation of an accumulation or depletion region of holes. A hole-depletion region is formed at the surface of the p-MOX with a higher valence band energy, while a hole-accumulation region is formed at the surface of the p-MOX with a lower valence-band energy (Figure 19c) [155]. During exposure to O_2_ and NH_3_, hole regions are modified, subsequently affecting the electrical conductivity.

The sensing results reported on p-type MOX towards NH_3_ are summarized in Table 1.

### 4.2. Oxidizing Gases

#### 4.2.1. NO_2_

NO_2_ is a poisonous and harmful gas that is damaging to the human respiratory system and the main cause of acid rain. NO_2_ is easily detectable by smell. Exposure to 4 ppm of NO_2_ anaesthetizes the nose, while increased concentrations may cause potential health risks. People with bronchitis or asthma are particularly sensitive to the presence of NO_2_, and lungs may become inflamed, leading to breathing difficulties [7]. Furthermore, several studies have also shown that NO_2_ exposure could cause lung damage in animals upon long-term exposure [156]. Accordingly, in 2010, the United States Environment Protection Agency (USEPA) fixed the air-quality standards for NO_2_ to 100 ppb for one-hour exposure and 53 ppb for one-year exposure (EPA-456/F-11-003) [157].

Many p-type MOX thin films, including Co_3_O_4_, CuO, and NiO, have been prepared to detect the NO_2_ gas in academic research [65,84,158,159,160,161,162]. However, the development of highly efficient, low-cost, reliable sensors that can operate at room temperature, with the ability to detect NO_2_ at concentrations below ppm or even at ppb levels, has yet to be achieved either in academic research or for practical applications.

In this scenario, Urso et al. reported the detection of sub-ppm levels of NO_2_ (140 ppb) at room temperature by nanoporous NiO thin film thanks to its porosity and the small size of NiO nanoparticles (30−50 nm) on the surface [163]. Additionally, two energetically different and independent adsorption sites (Langmuir sites and auxiliary sites) were identified as contributors to the sensing mechanism shown in Figure 19 [163]. In Brief, “Langmuir sites” are responsible for the short response time and long recovery time. “Auxiliary sites” have long response times and short recovery times. However, both sites contribute to NO_2_ detection at room temperature. Auxiliary sites become relevant only above an NO_2_ concentration threshold related to the full coverage of Langmuir sites. However, Langmuir sites are active at the optimal working temperature (150 °C) due to the favored NO_2_ desorption. Benedict et al. demonstrated the importance of slightly preferred orientation of the crystal structure and the higher porosity of the film toward the excellent NO_2_ sensing performance. In this case, short response and recovery times of 30 s and 45 s, respectively, were achieved towards 3 ppm NO_2_ at room temperature due to the aforementioned facts [164]. Subsequently, Al-Jumaili et al. demonstrated the significance of higher surface area toward NO_2_ sensing. In this study, nanoparticles of CuO thin film with higher surface area showed excellent sensing performance toward detecting NO_2_ at RT [161]. Furthermore, this study showed the possibility of increasing the response by 77–90% by improving the crystallite size during annealing.

MOX sensors typically work at elevated temperatures (thermoactivation) to activate the adsorption of gases on the surface of MOX. This results in an increase in the power consumption of the sensor. Therefore, “photoactivation”, which uses light instead of temperature to activate the surface reaction at low temperatures, has been proposed. Photoactivation can enhance the sensor’s kinetics, such as by increasing the speed of recovery of the sensors. Therefore, aside from functionalization, bulk doping, and heterostructures, photoactivation plays a key role in enhancing sensing performances of conductometric sensors. In this context, Katarzyna et al. studied the effect of UV irradiation on the response of a nanoporous NiO sensor towards NO_2_ concentrations ranging from 2 ppm to 16 ppm [165]. When the sensors was operated at 150 °C, the response increased approximately five times under UV irradiation (wavelength of 275 nm) compared with the response at dark measurement. Moreover, the limit of detection was 664 ppb and 525 ppb in dark and under UV illumination, respectively. This is attributed to the photo-generated electron–hole pair mechanism after light irradiation [166,167]. Most importantly, the surface reaction of nanoporous NiO towards NO_2_ and acetone were investigated by in situ FTIR measurements. The repeatability and selectivity towards NO_2_ can be explained by the formation of surface nitro species and nitrate ions (${\mathrm{NO}}_{3}^{-}$), as well as interband transitions during the adsorption of NO_2_ under illumination. The nitro and nitrate species are chemisorbed onto the sensor surface and displace oxygen, leading to an increase in electron extraction and a further reduction in sensor resistance. In contrast, the adsorption of acetone results in irreversible decomposition into formate and methoxy species, which cover the surface and limit gas sensing.

Usually, morphology of the synthesized materials, oxygen vacancies, grain size, and nature of the gas significantly govern NO_2_ gas sensing phenomena [168]. Hence, it would be useful to discuss how these facts influence the response. Khot et al. demonstrated that more porous and rougher surface morphology significantly improves NO_2_ sensing efficiency [160]. In this study, different samples of CuO thin films were grown by varying the precursor concentration in the spray-pyrolysis method at 350 °C. Different precursor concentrations produced different morphologies with a more or less porous nature, resulting in a higher response to 5 ppm NO_2_ of 56.23% in the roughest and most porous CuO thin films. On the other hand, spray duration in the spray-pyrolysis method influences the surface roughness. For example, NiO thin films prepared by Gomaa et al. showed the dependence of the surface roughness on the spray-pyrolysis duration and hence the hindering of the response towards NO_2_ [84]. In this study, NiO thin films prepared with a spray duration of 5 min showed the highest response of 57.3% towards 20 ppm NO_2_ at 200 °C, compared to the other samples with longer spray durations of 10, 15 and, 20 min. This effect was ascribed to a higher surface roughness. Additionally, Nalage et al. showed the effect of surface morphology and the number of vacant sites the gas adsorption [169]. In this case, a uniform nanocrystalline interconnected and porous structure (with an average particle size ranging from 30 to 50 nm) offered an enhanced surface and high NO_2_ adsorption rate. Moreover, a higher number of vacant sites in the film further improved gas adsorption, resulting in a lower recovery time.

The elemental doping of p-type MOX thin films is an interesting way to change not only the electrical properties but also the morphology of the oxide, thereby modifying the sensing performance. For example, Al doping has been found to cause a reduction in crystallite size, resulting in agglomerated grains, which increases grain boundaries and in turn increases NO_2_ adsorption. For instance, a lower amount of Al (5%) doping on NiO resulted in higher sensing response towards 1 ppm of NO_2_ at an operating temperature of 200 °C [162]. Doping with a higher concentration into to the host material could change the electrical-conductance type [170,171].

**Figure 20:**
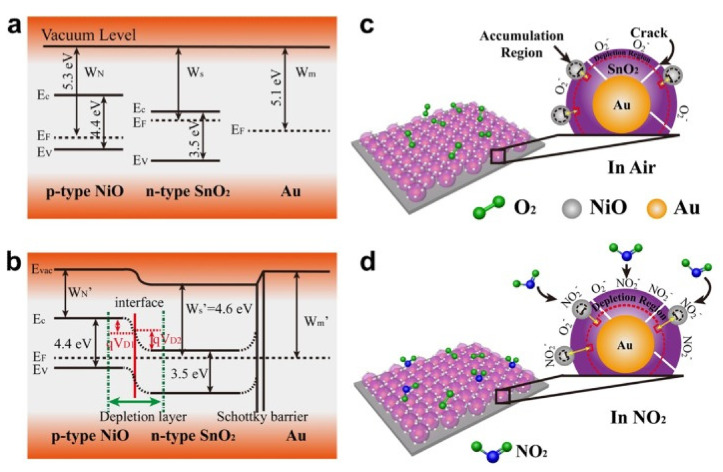
Schematics showing the NO_2_ sensing mechanism. (**a**) Schematic diagram of the energy-band configurations for NiO, SnO_2_, and Au. (**b**) Energy—band diagram of Au/SnO_2_/NiO heterojunction. (**c**,**d**) Schematic model for the Au/SnO_2_/NiO sensor exposed in air and NO_2_, respectively. The outside part of SnO_2_ indicated by red dashed lines is the depletion region. The black dashed lines in NiO show the accumulation region. White narrow strips indicate the existing cracks in SnO_2_ formed during thermal annealing. Reprinted with permission from [173].

Composite and heterostructure-based NO_2_ sensors should not be ignored. For instance, Zhang et al. demonstrated a response of 26.8% toward 5 ppm NO_2_ at RT on a reduced graphene oxide rGO/Co_3_O_4_ hybrid sensor [158]. The good sensing properties were attributed to larger specific surface area, more chemisorbed oxygen species, and a coupling effect between Co_3_O_4_ and graphene in the hybrid sensor. Besides, the formation of a pn heterojunction has attracted special attention as a promising approach to improve NO_2_ sensing performance of p-type MOX. These heterojunctions can enhance the response of gas sensors by changing the space-charge layer’s width between p-type and n-type MOX [172]. Accordingly, Wand et al. demonstrated a p-type conducting behavior of SnO_2_/NiO thin films towards 5 ppm of NO_2_ at 500 °C. In this case, the SnO_2_/NiO heterojunction was prepared by magnetron sputtering using an SnO_2_/NiO composite target. Moreover, Au NPs were used to enhance response. In this case, the higher response (180 towards 5 ppm) of the prepared Au/SnO_2_:NiO sensors compared to that of the SnO_2_:NiO (2.5 towards 5 ppm) sensor was attributed both to the catalytic role of the Au NPs and the design of heterojunctions in Au/SnO_2_/NiO thin film, with a relatively high work function of 4.56 eV, as shown in Figure 20 [173].

**Figure 21:**
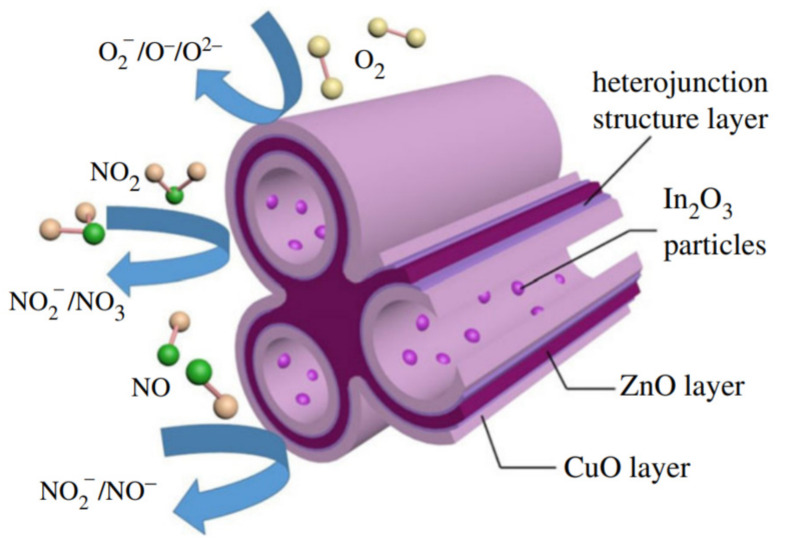
Schematic diagram of the sensing mechanism of a 1 wt % In_2_O_3_–CuO/ZnO sensor. Reprinted with permission from [174].

Subsequently, Li et al. proposed the decoration of composite CuO/ZnO with In_2_O_3_ to achieve fast response times [174]. In this study, the prepared complex composite structure (1 wt % In_2_O_3_–CuO/ZnO) showed a response time of 7 s. Moreover, the sensor showed a response of 82% towards 100 ppm of NO_2_ at RT. However, the sensing performances can be ascribed to different factors. First, the fluffy, porous structure promotes and enhances gas diffusion and surface reactions, which lead to a faster response. Moreover, the np heterojunction between CuO and ZnO plays an essential role in enhancing the sensor response and selectively. In this case, CuO has a lower Fermi level than ZnO. Therefore, an accumulation layer at the CuO/ZnO interface is formed. The increase in electrons on the surface of ZnO can facilitate the adsorption of oxygen molecules and a decrease in resistance. Furthermore, the catalytic activity of In_2_O_3_ nanoparticles accelerates the dissociation of oxygen molecules and causes a spillover of the adsorbed oxygen ions on the composite surface. Lastly, the higher amount of adsorbed oxygen ions provides more sensing sites. A schematic of the proposed sensing mechanism in this composite is shown in Figure 21 [174]. The general reactions that occur between NO_2_ and the surface of MOX at room temperature can be described by Equations (8) and (9).

(8) $${\mathrm{NO}}_{2}+e^{-}\to {\mathrm{NO}}_{2}^{-}$$

(9) $${\mathrm{NO}}_{2}+O_{2}^{-}+2e^{-}\to {\mathrm{NO}}_{2}^{-}+2O^{-}$$

The sensing results reported on p-type MOX towards NO_2_ are summarized in Table 1.

#### 4.2.2. CO_2_

Carbon dioxide (CO_2_) is another oxidizing gas that is usually emitted by two means. Natural emissions of CO_2_ occur in Earth’s atmosphere due to the carbon cycle. Human-made (or anthropogenic) CO_2_ emissions are mainly due to the combustion of fossil fuels in transport, industry, and electricity. However, the rapid increment of CO_2_ emission has a significant and direct impact on human health and the environment. For instance, an elevated indoor CO_2_ level causes significant symptoms for humans: reduction in mental concentration, fatigue, headache, and dizziness [175]. Thus, it is extremely important to detect and quantify CO_2_. On the other hand, there are few references in the literature on p-type MOX CO_2_ sensors.

Composite semiconductors with a p-n junction were used to improve CO_2_ sensing performances by reducing the operating temperature and increasing response [176]. Chapelle et al. achieved an excellent response in CuO/CuFe_2_O_4_ composite thin films towards CO_2_ at a working temperature of 250 °C, this study proves the significant role of thickness in sensing properties. In short, a bilayer p-CuO/n-CuFe_2_O_4_ structure was fabricated with a CuO layer thickness of 50 nm and 100 nm. CuO (300 nm thick) exhibited higher response (15%) and quasi-long recovery time (150 s) and operating temperature (370 °C) in comparison to CuO (50 nm thick), which exhibited a high response (40%), fast response (55 s), fast recovery time (8 s), and lower operating temperature (250 °C) [177]. Moreover, the authors demonstrated a 25% improvement in the response towards CO_2_ after functionalization of CuO (50 nm)/CuFe_2_O_4_ structure with silver, compared to a nonfunctionalized thin-film composite. On the other hand, surface morphology can improve the response towards CO_2_. For example, Joshi et al. showed a higher response (13.68) on porous SnO_2_–Co_3_O_4_ composites (prepared by mixing solution SnO_2_; Co_3_O_4_ by 1;2 volume) towards 500 ppm of CO_2_ at 30 °C compared to that on SnO_2_ (0.04) or Co_3_O_4_ (0.07). The enhanced response of the material was ascribed to the band configuration of SnO_2_/Co_3_O_4_ [178]. Subsequently, Tanvir et al. introduced a combination of organic binder and peroxide, such as ZnO_2_, to improve sensing of CuO. Indeed, the mixture led to the formation of a CuO/ZnO_2_ (10:1 NPs layer) composite. The developed sensor was investigated at several operating temperatures, ranging from 20 °C to 600 °C, at various relative-humidity (RH) levels, such as 0%, 30%, 60%, and 80%, towards CO_2_ concentrations ranging from 400 to 4000 ppm. The film’s resistance was reported to increase in the presence of moisture due to the formation of carbonate structures (CuO_x_(CO_3_)y(OH)_z_) on the surface of copper oxide and grain boundaries, which act as potential barriers. Additionally, the response of the sensor was decreased when the humidity level exceeded 30%. In contrast, the change of accumulation film in dry air was responsible for the resistance drop. However, the optimal parameters were a 300 °C operating temperature and 30% RH with CuO/ZnO_2_ (10:1 NPs layer) to detect 1000 ppm CO_2_ at a response rate of 13% [179].

**Figure 22:**
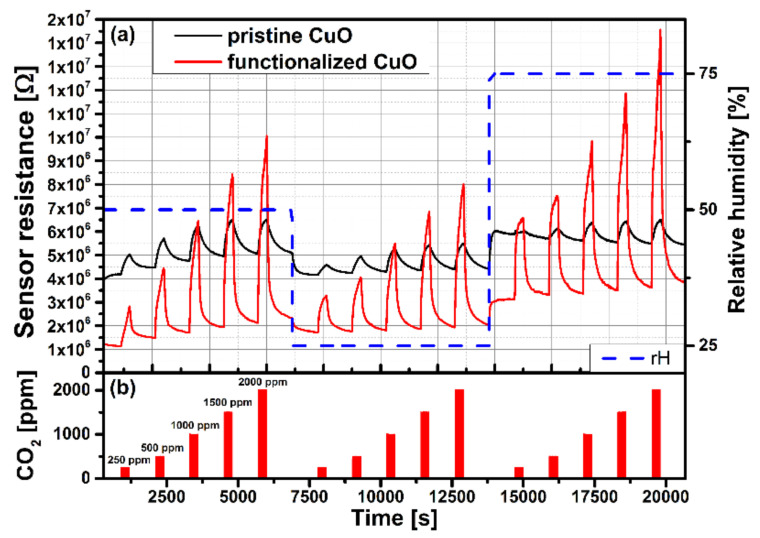
(**a**) Carbon dioxide measurement of pristine and Au-functionalized CuO gas sensors at an operation temperature of 300 °C and relative humidity levels of 25%, 50%, and 75%. (**b**) CO_2_ gas pulses: 250 ppm, 500 ppm, 100 ppm, 1500 ppm, and 2000 ppm. Reprinted with permission from [175].

Relative humidity is another factor that affects the performance of sensors. Exploring sensors that can operate in an environment with a high relative humidity level (>40%) may provide an opportunity to develop sensors for breath-analysis applications. Accordingly, Teubenbacher et al. investigated the excellent response of Au-functionalized CuO thin films towards 2000 ppm of CO_2_ in the presence of 50% RH (Figure 22). The Au-functionalized CuO sensor had an improved performance 13 times better than that of CuO thin film [175]. In this study, enhancement was achieved by the additional active sites on the interface of the metal oxide due to gold nanoparticles and the formation of malachite, which encourages CO_2_ adsorption. However, the sensor’s performance was hindered by the dissociation of formed malachite or azurite when the operating temperature exceeded 300 °C. The general reaction that occurs between CO_2_ and the surface of MOX at room temperature can be described by Equation (10). The sensing results reported on p-type MOX towards CO_2_ are summarized in Table 1.

(10) $${\mathrm{CO}}_{2}+2O_{2}^{-}\to 2{\mathrm{CO}}_{3}^{2-}$$

#### 4.2.3. O_3_

Ozone (O_3_) is a strong oxidizing agent that is useful for both positive and negativemeans. In many industries, ozone is used for water treatment and gas purification, and it is also used to inactivate the microbial content of foodstuffs, including bacteria, fungi, and viruses. However, according to the World Health Organization (WHO), the air quality guideline for ozone is set at ∼50 ppb (100 μg/m^3^) for a daily maximum 8h mean. Exposure to 0.1–1 ppm causes headaches, burning eyes, and irritation to the respiratory passages [180] (WHO Air quality guidelines for particulate matter, ozone, nitrogen dioxide and sulfur dioxide Global update 2005, WHO/SDE/PHE/OEH/06.02). However, the reported work on O_3_ sensing is limited. Surface morphology is the key factor that governs the response of thin-film gas sensors. In this case, the grain size is the most significant factor. The sensing performances of CuO thin films toward ozone vary with grain size, as demonstrated by Bejaoui et al. Their study demonstrated that a grain size of 25 nm is the optimum size for the detection of ozone (0.3 ppm) at 250 °C [181]. Furthermore, Kumar et al. demonstrated that ozone sensing can be improved by reducing the crystalline dimension of NiO thin films. In their study, NiO thin film annealed at 200 °C demonstrated excellent sensing performance (12.3) toward ozone (70 ppb) at a working temperature of 200 °C [182]. Additionally, Paralikis et al. investigated the detection of O_3_ at RT. In this study, Al-doped NiO (NiO:Al) thin films were grown by co-sputtering Ni and Al with two stubs on the Ni target by varying the plasma-oxygen content. The films prepared with an O_2_ content of 4% demonstrated high response (3.18%) towards 60 ppm of ozone at room temperature. In addition, this study demonstrated that the detection limit can be reduced to 10 ppb by increasing the operating temperature to 80 °C [183]. The sensing results reported on p-type MOX towards O_3_ are summarized in Table 1. The general reaction that occurs between O_3_ and the surface of MOX at room temperature can be described by Equation (11).

(11) $$O_{3}+e^{-}\to O^{-}+O_{2}$$

## 5. Future Trends

Nanowires, nanorods, nanobelts, nanofibers, etc., are one-dimensional (1D) nanostructures that have recently gained immense attention as potential morphologies for the enhancement of chemical sensing performances. Nanowires in particular have been the subject of considerable attention in the last few years. Several characteristics of p-type MOX nanowires, such as high aspect ratios, high surface-area-to-volume ratios, high crystallinity, high thermal stability, high carrier-charge transport, high chemical stability, and electronic compatibility, make them a promising choice for the fabrication of chemical sensors. P-type MOX nanowires may overcome one of the primary challenges of gas-sensor devices based on MOX thin films, i.e., coalescence of the grains affecting long-term stability at high operating temperatures. Moreover, nanowires with a diameter below 25 nm and a length of several micrometers could provide high surface area and improved surface chemistry, as well as high carrier-charge transport due to their one-dimensional structure. Indeed, a few problems remain, such as a lack of selectivity and high working temperatures, which require surface or bulk modification order to be resolved.

Among the methods discussed in this review, thermal oxidation is the most suitable method for the fabrication of 1D p-type MOX, particularly nanowires. The sol–gel method can be applied to the growth of these nanostructures; however, precise control and the choice of surfactants are necessary, whereas electrodeposition can be used to prepare 1D MOX nanotubes and nanowires at low temperatures. In contrast, growing MOX nanowires by magnetron sputtering and MBE remain a challenging task. Further, there are famous techniques that can be used to produce p-type MOX nanowires, including the evaporation-condensation method (using the vapor–solid–liquid (VLS) mechanism) and the solvothermal method.

Most importantly, reports usually focus on gas sensors based on p-type nanowire networks instead of p-type MOX single nanowires, which are still under development. Gas sensors based on single or individual nanowires are characterized by the possibility of exploiting the self-heating effect, which helps to reduce the power consumption of the final device. Nevertheless, due to the inherent difficulties associated with working at the nanoscale, the separation and characterization of individual nanowires are not straightforward tasks and require further research and development.

The possibility of integrating 1D nanostructure within thin films forming heterostructures with different morphologies may allow new strategies to improve chemical sensing performances and tackle existing challenges.

In addition to metal-oxide nanowires, extensive research has been conducted on 2D nanomaterials in recent years. 2D nanomaterials, such as transition-metal dichalcogenides (2D TMDC), nanosheets, or nanoflakes, are considered a new class of nanomaterials for chemical-sensor applications and are estimated to outperform carbon-based (i.e., graphene, carbon nanotube, etc.) chemical sensors.

The most widely used technique for preparing 2D TMDC is chemical vapor deposition (CVD). CVD techniques are normally used to prepare bulk 2D TMDC. CVD growth is commonly accompanied by specific exfoliation methods (mechanical exfoliation, such as the Scotch-tape method; or liquid-phase exfoliation, such as sonication electrochemical exfoliation), which can separate the layers and yield to a mono- or multilayered 2D TMDC. MBE is another method that can be used to grow 2D TMDC, and it offers ultimate control down to the atomic layer. Additionally, wet-chemical and solvothermal techniques can be used to prepare 2D TMDC structures. These techniques can produce different morphologies and assembled structures of TMDC. The chemical/surface interaction is controlled by absorption energy and the amount of transferred carrier charge. Thanks to their lateral size and surface-to-volume ratio, as well as their rich surface chemistry, TMDC nanosheets are ideal candidates for chemical-sensing applications. However, challenges such as large-scale mass production and the thermal instability of 2D materials, as in the case of metal chalcogenides, are the drawbacks that must be overcome for practical application. A mono- or few-layer TMDC is characterized by an ultra-large specific area, excellent carrier transport, and high mechanical flexibility. TMDC materials such as MoTe_2_, MoS_2_, MoSe_2_, WS_2_, and WSe_2_ have demonstrated high sensitivity to several chemical compounds. A layered structure provides a large, active surface for interactions with chemical compounds, point defects, and edges that play a crucial role in gas-sensing applications. Due to the incorporation of oxygen (oxidation), TMDCs are not stable under high operating temperatures. However, mono- or few-layer TMDC nanosheets demonstrate a high response at low temperatures (below 200 °C), which opens up possibilities for portable applications. They are also mechanically stable, which makes them suitable for flexible, wearable sensor platforms.

Recently research has found that the use of hybrid structures, such as 2D TMDC/MOX nanowires and 2D TMDC/MOX thin films, could be an effective strategy for boosting chemical-sensing performance. In addition to increasing the specific area and the number of active sites for chemical adsorption, these combinations result in different heterojunctions (pn, nn, and pp). These hybrids may meet the actual challenges of chemical sensors.

## 6. Conclusions

The synthesis of p-type MOX thin films, such as CuO, NiO, Co_3_O_4_, and Cr_2_O_3_ and their efficiency in chemical-sensor applications were reviewed. Two approaches have been used for the synthesis of p-type MOX thin films: vapor-phase and liquid-phase methods.

PVD precisely controls the thickness, crystal structure, and microstructure of thin films, despite a high cost. Several methods have reported interesting results; magnetron sputtering, thermal evaporation, thermal oxidation, and molecular-beam epitaxy (MBE) are among the most commonly investigated. Despite the high cost of magnetron sputtering, it is the most commonly used method for growing metal-oxide thin films with high control over stoichiometry and thickness, as well as the ability to fabricate MOX heterostructures and possible growth at low temperatures. Due to the ultra-high vacuum, MBE is suitable for growing epitaxial single-crystal thin films with high purity and precise control of the thickness. However, this process is expensive and requires predefined substrates to fulfill lattice matching.

On the other hand, thermal evaporation and thermal oxidation are low-cost methods used to grow p-type MOX thin films with limited control over thickness and high growth temperatures. CVD techniques offer high control over morphology and defects, along with the formation of heterojunctions; unfortunately, harmful byproducts are formed. Parameters such as oxygen content, thickness, and thermodynamic conditions, including temperature and pressure, have a direct effect on the crystallinity, grain size, stoichiometry, defects, and porosity of MOX thin films, which are factors that, in turn, determine the sensing characteristics.

Liquid-phase synthesis methods enable routes for metal-oxide thin films and their novel low-temperature applications. Among these synthesis methods, sol–gel-assisted spin-and-dip coating, electrodeposition, and spray pyrolysis have been widely employed. Further attention must be paid to understanding the role of each synthesis step with regards to large-scale applications because of limitations, such as mixing, mass and heat transfer, and batch-to-batch variations in the final products.

Several p-type MOX thin films have been prepared by various synthesis methods, and their different sensing properties towards specific compounds have been demonstrated, irrespective of the synthesis method. The most dominant features are “thickness” and “grain size”, which impact the resistance of thin films, as well as grain-boundary resistance. P-type MOX thin films have been prepared by controlling nucleation and growth processes to detect reducing (VOCs, H_2_, and NH_3_) and oxidizing gases (NO_2_, CO_2_, and O_3_). CuO and NiO are the most investigated p-type MOX thin films, and have shown high responses, whereas Co_3_O_4_ and Cr_2_O_3_ have been used to make composite materials due to their excellent catalytic effects to further improve the adsorption rate and sensing capabilities. After all, p-type MOX show high responses and acceptable long-term stability, along with stable responses at high humidity levels, although they suffer from lack of selectivity. Most surprisingly, many p-type MOX gas-sensor devices are capable of detecting ammonia at room temperature. To explain this finding, more clarification is needed.

In this review, possible ways to improve the sensing properties of MOX thin films were discussed—in particular, selectivity. Among these, there are spillover effects and electronic sensitization caused by metal functionalization, bulk doping and mixed MOX or composite-structure-based p-type MOX form p-p junctions or p-n junctions. These strategies increase the number of active sites, enhance oxygen-gas adsorption, affect the cation vacancies in p-type MOX, and modify the hole-accumulation layer due to carrier-charge transfer. Furthermore, p-type MOX thin films have been proven capable of sensing many compunds, such as ethanol, acetone, formaldehyde, propane, isopropanol, H_2_, NH_3_, and NO_2_. However, limitations in performance were observed in the detection of CO_2_ and O_3_.

## Figures and Tables

**Table 1:** Sensing performance of p-type MOX towards reducing and oxidizing gases.

Material	Synthesis Method	Operating Temperature (°C)	Gas/ppm	Response	Response/Recovery Time (s)	LOD	Long-Term Stability/Reproducibility	Ref.
NiO	Photolithography-assisted spin coating	350	Ethanol/5 ppm	1.27 ^a^	80 s/120 s	NA	NA/NA	[110]
NiO	Magnetron sputtering	300	Ethanol/5 ppm	5 ^b^	167 s/99 s	0.1 ppm	NA/NA	[111]
CuO	Wet chemical method	300	Acetone/50 ppm	2 ^b^	NA/NA	NA	180 days/3 cycles	[112]
Co_3_O_4_	Spray pyrolysis	RT	Acetone/50 ppm	235 ^a^	6 s/4 s	1 ppm	60 days/5 cycles	[113]
Al-doped NiO	Magnetron sputtering	RT+UV irradiation	Methane/NA	58% ^b^	1373 s/249 s	NA	NA/3 cycles	[118]
CuO-Ga_2_O_3_	Magnetron sputtering	300	Acetone/1.25 ppm	1.35 ^a^	187 s/525 s	NA	NA/NA	[120]
n-ZnO/p-NiO	Wet-chemical-route-assisted spin coating	300	Acetone/500 ppm	NA	13 s/18 s	NA	NA/NA	[122]
PtO_2_-functionalized CuO	Two-step method	180	n-butanol/100 ppm	12 ^a^	2.4 s/9.2 s	NA	30 days/6 cycles	[128]
NiO/SnO_2_	Magnetron sputtering	250	Ethanol/100 ppm	7.9 ^NA^	15 s/100 s	100 ppb	NA/4 cycles	[130]
NiO	Magnetron sputtering	250	H_2_/1%	416 ^b^	7 s/153 s	≤50 ppm	NA/NA	[134]
NiO	Spray pyrolysis	300	H_2_/500 ppm	55 ^b^	38 s/41 s	NA	NA/NA	[136]
Cr_2_O_3_	Magnetron sputtering	400	H_2_/2000 ppm	40%^NA^	NA	NA	NA/NA	[137]
Co_3_O_4_/SnO_2_	Soak-calcination method	300	H_2_/50 ppm	30% ^b^	NA	NA	NA/24 cycles	[140]
Al_2_O_3_/CuO	ALD	350	H_2_/100 ppm	80% ^b^	20.8 s/59.9 s	NA	70 days/3 cycles	[142]
Au-functionalized NiO	Magnetron sputtering	125	H_2_/500 ppm	1 ^b^	15 min to ∼5 min/NA	2 ppm	NA/NA	[145]
NiO	Magnetron sputtering	140	H_2_/10000 ppm	14 ^b^	3 min/NA	NA	NA/NA	[141]
Pd-functionalized CuO	Magnetron sputtering	300	H_2_/1000 ppm	3 ^a^	10 s/50 s	NA	21 days/NA	[148]
CuO/SnO_2_	Magnetron sputtering	RT	NH_3_/100 ppm	3353 ^a^	266 s/35 s	NA	6 months/5 cycles	[150]
CuO/SnO_2_/ZnO	Magnetron sputtering	RT	NH_3_/100 ppm	2057 ^a^	294 s/47 s	NA	6 months/5 cycles	[151]
MoS_2_/CuO	Magnetron sputtering	RT	NH_3_/100 ppm	47% ^b^	17 s/26 s	NA	70 days/15 cycles	[152]
Cr_2_O_3_/CuO	Magnetron sputtering	RT	NH_3_/25 ppm	77% ^b^	11 s/14 s	14.1 ppm	NA/6 cycles	[154]
CuO	Spray pyrolysis	200	NO_2_/ 5 ppm	56.23% ^b^	20.57 s/235.2 s	NA	N/A	[160]
NiO	Sol–gel spin coating	200	NO_2_/ 20 ppm	57.3% ^b^	20 s/498 s	NA	20 days/	[169]
Al/NiO	RF sputtering	200	NO_2_/ 1 ppm	576 ^b^	2160 s/3300 s	NA	365 days	[162]
2.4% rGO-Co_3_O_4_	Facile two-step method	RT	NO_2_/ 5 ppm	26.8% ^b^	210 s/60 s	50 ppb	20 days/5 cycles	[158]
Au-functionalized CuO	Electron-beam lithography, thermal evaporation	300	CO_2_/2000 ppm	365% ^b^	258 s/264 s	N/A	14 days/NA	[175]
CuO/CuFe_2_O_4_	RF sputtering	250	CO_2_/5000 ppm	40 ^b^	3300 s/480 s	NA/NA	NA/NA	[177]
SnO_2_–Co_3_O_4_	Sol–gel spin coating	30	CO_2_/2000 ppm	13.68 ^b^	2 s/12 s	NA	NA/NA	[178]
NiO	Chemical-bath deposition	RT	NO_2_/ 140 ppb	3.5 ^b^	75 s/174 s	20 ppb	30 days/NA/NA	[163]
NiO	Microwave-assisted deposition	RT	NO_2_/ 3 ppm	4991% ^b^	30 s/45 s	200 ppb	NA/NA	[164]
NiO	Sol–gel spin coating	200	NO_2_/ 200 ppm	23.3 ^b^	20 s/498 s	NA	30 days/NA	[169]
In_2_O_3_–CuO/ZnO	Electroplating and chemical plating	RT	NOx/ 100 ppm	82 ^b^	7 s/NA	1000 ppb	6 months/NA	[174]
Al-doped NiO	RF sputtering	150	O_3_/80 ppb	5.17% ^b^	189.6 s/243.6 s	10 ppb	NA/NA	[183]

Note: ^(a)^ Response = (G, I or R _air_/(G, I or R)_gas_ or = (G, I or R)_gas_/(G, I or R)_air_; ^(b)^ Response = (ΔG/G) or (ΔI/I) or (ΔR/R); NA = not available.

## Data Availability

Not applicable.
